# Hepatic small extracellular vesicles promote microvascular endothelial hyperpermeability during NAFLD via novel-miRNA-7

**DOI:** 10.1186/s12951-021-01137-3

**Published:** 2021-11-27

**Authors:** Rui Zuo, Li-Feng Ye, Yi Huang, Zi-Qing Song, Lei Wang, Hui Zhi, Min-Yi Zhang, Jie-Yi Li, Li Zhu, Wen-Jing Xiao, Hong-Cai Shang, Yang Zhang, Rong-Rong He, Yang Chen

**Affiliations:** 1 Department of Pharmacology, School of Pharmaceutical, Guangzhou University of Chinese Medicine, 232, Waihuan East Road, Guangzhou Higher Education Mega Center, Panyu District, Guangzhou, 510000 China; 2 Department of Stomatology, The First Affiliated Hospital, The School of Dental Medicine, Jinan University, Guangzhou, China; 3 Key Laboratory of Chinese Internal Medicine of Ministry of Education and Beijing, Dongzhimen Hospital Affiliated to Beijing University of Chinese Medicine, 5 Hai Yun Cang, Dongcheng District, Beijing, 100700 China; 4 Department of Pharmacological and Pharmaceutical Sciences, College of Pharmacy, University of Houston, 4849 Calhoun Road, Houston, TX 77204-5037 USA; 5 Guangdong Engineering Research Center of Chinese Medicine and Disease Susceptibility, Jinan University, 601, West Huangpu Road, Guangzhou, 510632 China

**Keywords:** Liver inflammation, Microvascular endothelial dysfunction, Inter-organ communication, Tight junction, NLRP3 inflammasome

## Abstract

**Background:**

A recent study has reported that patients with nonalcoholic fatty liver disease (NAFLD) are more susceptible to coronary microvascular dysfunction (CMD), which may predict major adverse cardiac events. However, little is known regarding the causes of CMD during NAFLD. In this study, we aimed to explore the role of hepatic small extracellular vesicles (sEVs) in regulating the endothelial dysfunction of coronary microvessels during NAFLD.

**Results:**

We established two murine NAFLD models by feeding mice a methionine-choline-deficient (MCD) diet for 4 weeks or a high-fat diet (HFD) for 16 weeks. We found that the NOD-like receptor family, pyrin domain containing 3 (NLRP3) inflammasome-dependent endothelial hyperpermeability occurred in coronary microvessels during both MCD diet and HFD-induced NAFLD. The in vivo and in vitro experiments proved that novel-microRNA(miR)-7-abundant hepatic sEVs were responsible for NLRP3 inflammasome-dependent endothelial barrier dysfunction. Mechanistically, novel-miR-7 directly targeted lysosomal associated membrane protein 1 (LAMP1) and promotes lysosomal membrane permeability (LMP), which in turn induced Cathepsin B-dependent NLRP3 inflammasome activation and microvascular endothelial hyperpermeability. Conversely, a specific novel-miR-7 inhibitor markedly improved endothelial barrier integrity. Finally, we proved that steatotic hepatocyte was a significant source of novel-miR-7-contained hepatic sEVs, and steatotic hepatocyte-derived sEVs were able to promote NLRP3 inflammasome-dependent microvascular endothelial hyperpermeability through novel-miR-7.

**Conclusions:**

Hepatic sEVs contribute to endothelial hyperpermeability in coronary microvessels by delivering novel-miR-7 and targeting the LAMP1/Cathepsin B/NLRP3 inflammasome axis during NAFLD. Our study brings new insights into the liver-to-microvessel cross-talk and may provide a new diagnostic biomarker and treatment target for microvascular complications of NAFLD.

**Graphical Abstract:**

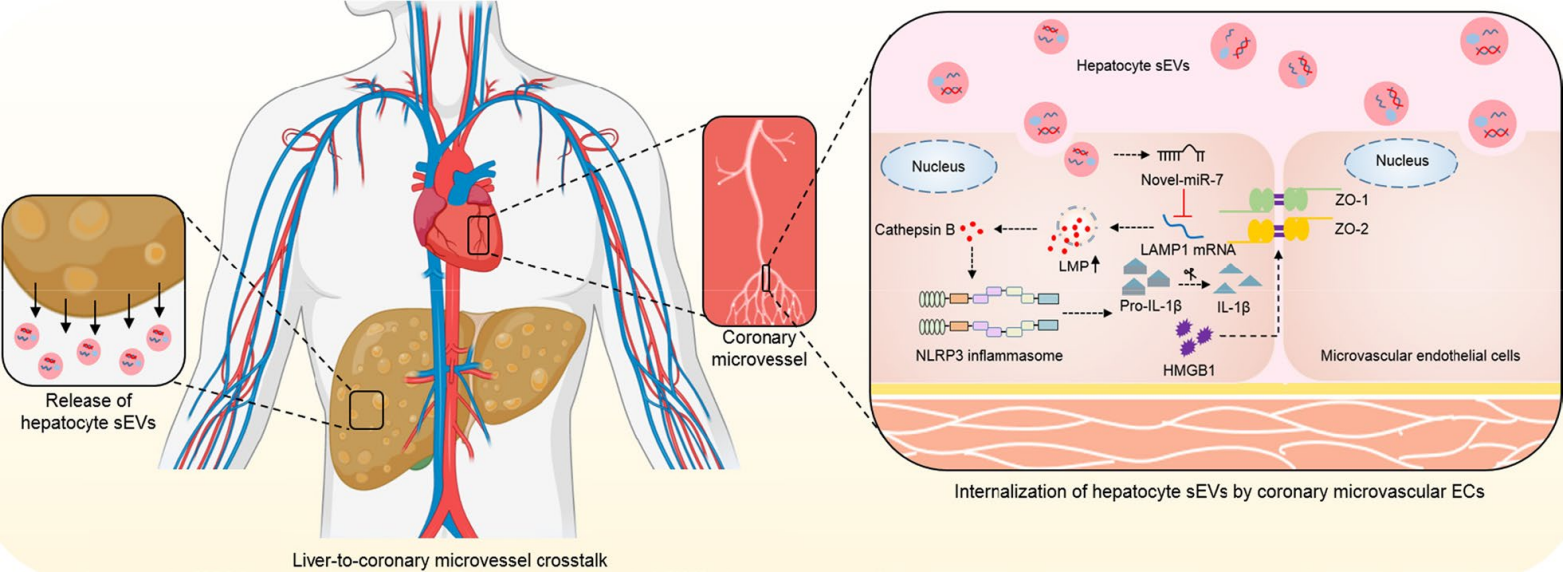

**Supplementary Information:**

The online version contains supplementary material available at 10.1186/s12951-021-01137-3.

## Background

Nonalcoholic fatty liver disease (NAFLD) is the most common chronic liver disease that comprises a wide spectrum ranging from simple steatosis to steatohepatitis [1]. Recent studies have reported that NAFLD patients are more susceptible to coronary microvascular dysfunction (CMD), which may predict major adverse cardiac events such as myocardial infarctions and heart failure [2, 3]. The integrity of coronary microvascular circulation is impaired in approximately 42.4% of patients with NAFLD, manifested as the abnormal coronary flow reserve [4]. Up to now, the causes of CMD during NAFLD remain undefined. Furthermore, very little research has gone into developing diagnostic biomarkers for CMD and therapeutic interventions to improve coronary microcirculatory function during NAFLD.

CMD is initiated from the chronic inflammatory in response to cardiovascular risk factors [5]. Continuous exposure to a pro-inflammatory environment contributes to the microvascular endothelial hyperpermeability, which could promote regional inflammatory infiltration and oedema with the latter further reduces coronary blood perfusion [6]. Endothelial tight junctions are crucial structures that restrict the transport of water, ions, and molecules across the endothelial monolayer through mediating paracellular permeability [7]. Reduction of the tight junction proteins zonula occludens (ZO)-1/2 promotes endothelial hyperpermeability and is regarded as the early onset of cardiovascular events [8, 9]. Previously studies have identified that the NOD-like receptor family, pyrin domain containing 3 (NLRP3) inflammasome activation is a significant mechanism for regulating tight junction disruption through mediating the release of high mobility group box 1 (HMGB1) [10–12]. NLRP3 inflammasome is known as a multiprotein complex that act as a platform for caspase 1-dependent proteolytic maturation and production of pro-inflammatory cytokines. However, the role of NLRP3 inflammasome in the development of microvascular endothelial hyperpermeability during NAFLD is still poorly understood.

In recent years, small extracellular vesicles (sEVs) have emerged as crucial regulators of organ-to-organ communication in both physiological and pathological conditions [13, 14]. SEV is a subpopulation of extracellular vesicles, with a diameter of 50–200 nm and functions as a paracrine effector by delivering bioactive cargos to recipient cells [15]. During the development of NAFLD, sEVs derived from damaged hepatocytes induce the occurrence of inflammation, fibrogenesis, and angiogenesis [16]. A recent study has revealed that steatotic hepatocyte sEV exhibit a pro-inflammatory effect and contributes to vascular atherogenesis by delivering microRNA(miRNA)-1 [17], suggesting the critical roles of sEV-contained miRNA cargo in regulating endothelial function.

In this study, we demonstrate that novel-miR-7-enriched hepatic sEV directly targets the lysosomal-associated membrane protein 1 (LAMP1)/Cathepsin B/NLRP3 inflammasome axis and induces microvascular endothelial hyperpermeability during NAFLD. We further determine that steatotic hepatocyte is a significant source of novel-miR-7-abundant sEVs, and genetic inhibition of novel-miR-7 profoundly improves endothelial permeability alternations. Our study revealed a critical role of novel-miR-7-contained hepatocyte sEVs in the inter-organ communication mechanism between the liver and coronary microvessel, and may contribute to the investigation of diagnostic biomarkers and novel therapeutic targets of microvascular complications of NAFLD.

## Results

### Endothelial permeability is enhanced in coronary microvessels of NAFLD mice

**Fig. 1:**
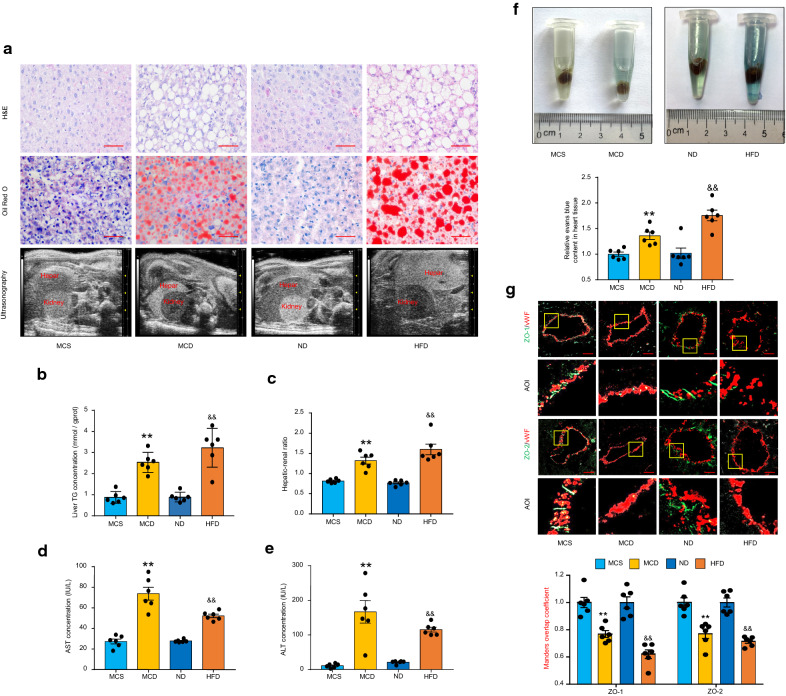
Endothelial permeability is enhanced in coronary microvessels of NAFLD mice. Mice were fed a MCD diet for 4 weeks or HFD for 16 weeks to induce NAFLD. **A** Representative images of liver H&E staining, oil red O staining and B-scan ultrasonographic imaging, n = 6 per group. Scale bar: 200 μm. **B** Liver triglyceride (TG) content, n = 6 per group. **C** The ratio of hepatic-renal echo-intensity, n = 6 per group. **D**, **E** Plasma levels of aspartate aminotransferase (AST) and alanine aminotransferase (ALT), n = 6 per group. **F** Representative images and the summarized data of Evans blue concentration in heart tissues, n = 6 per group. **G** Representative fluorescent confocal images of ZO-1/2 (green) with vWF (red) and the summarized data of the Manders overlap coefficient of ZO-1/2 over vWF. The area of interest (AOI) is selected for higher magnification, n = 6 per group. Scale bar, 20 μm. Data are expressed as the mean ± SEM. Statistics: Student *t*-test, **P < 0.01 vs. the MCS group; ^&&^*P* < 0.01 vs. the ND group

To establish murine NAFLD models, we fed male C57BL/6 N mice with a MCD diet for 4 weeks or HFD for 16 weeks. Results showed that both the MCD diet and HFD administration induced the anticipated features of NAFLD with severe hepatic steatosis (Fig. 1A) and intrahepatic triglyceride accumulation (Fig. 1A, B), as well as elevated hepatic-renal ratio (Fig. 1A, C) and aminotransferase activities (Fig. 1D, E). It is worth noting that mice fed a HFD diet gained body and liver weight (Additional file 1: Fig. S1A, B) as well as developed dyslipidemia (Additional file 1: Fig. S1C–E). In contrast, MCD-diet fed mice did not mimic such metabolic syndromes associated with human NAFLD. Subsequently, we estimated the permeability changes of coronary microvessels in both MCD and HFD-induced NAFLD mice by measuring Evans blue leakage. As shown in Fig. 1F, the darker blue suggested that the coronary microvessels of NAFLD mice were more permeable than control mice. Furthermore, the relative fluorescence intensity ratios of ZO-1/2 and the von Willebrand factor (vWF), a specific endothelial marker, showed that the integrity of ZO-1/2 was markedly decreased in the endothelium of coronary microvessels in both MCD and HFD groups (Fig. 1G). Taken together, these results indicate that endothelial barrier dysfunction occurs in coronary microvessels during NAFLD and does not rely on the metabolic features.

### NLRP3 inflammasome mediates endothelial hyperpermeability of coronary microvessels in NAFLD mice

**Fig. 2:**
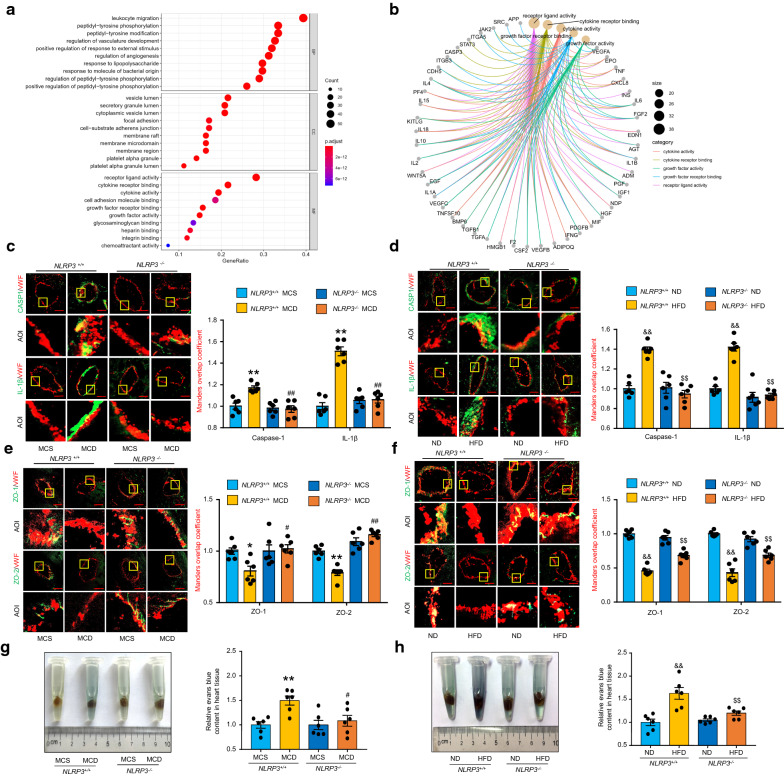
NLRP3 inflammasome mediates endothelial hyperpermeability of coronary microvessels in NAFLD mice. The human targets of microvascular hyperpermeability was gathered from the GeneCards database, and the Gene Ontology (GO) analysis was performed to analyze the main function of target genes. *NLRP3*^+*/*+^ and *NLRP3*^*−/−*^ mice were fed a MCD diet for 4 weeks or HFD for 16 weeks to induce NAFLD. **A**, **B** GO enrichment analysis of the human targets of microvascular hyperpermeability. The size of the circles represents the number of child GO term. The color represents the significance of the enrichment or the category of molecular function. BP: Biological Process, CC: Cellular Component, MF: Molecular Function. **C**–**F** Representative fluorescent confocal images of cleaved-caspase-1(CASP1), IL-1β, and ZO-1/2 (green) with vWF (red) and the summarized data of the Manders overlap coefficient. The area of interest (AOI) is selected for higher magnification, n = 6 per group. Scale bar, 20 μm. **G**, **H** Representative images and the summarized data of Evans blue concentrations in heart tissues, n = 6 per group. Data are expressed as the mean ± SEM. Statistics: One-way ANOVA, *P < 0.05, **P < 0.01 vs. *NLRP3*^+*/*+^ mice fed with MCS diet; ^#^*P* < 0.05, ^##^*P* < 0.01 vs. *NLRP3*^+*/*+^ mice fed with MCD diet; ^&&^*P* < 0.01 vs. *NLRP3*^+*/*+^ mice fed with ND; ^$$^*P* < 0.01 vs. *NLRP3*^+*/*+^ mice fed with HFD

To explore the upstream mechanism of microvascular permeability changes during NAFLD, the human targets of microvascular hyperpermeability from GeneCards dataset were used to perform the Gene Ontology (GO) enrichment analysis. According to the result of Molecular Function (MF) enrichment, the cytokine activity and cytokine receptor binding are important in the development of microvascular hyperpermeability (Fig. 2A). In order to find out the key mediators, we screened the common genes from these two pathway (Fig. 2B). Among them, the common gene with a correlation score ≥ 4 was considered to be closely related to microvascular hyperpermeability. Subsequently, we performed KEGG pathway enrichment analysis to reveal the relevant signaling pathway (Additional file 1: Fig. S12). Results showed that the target genes are mainly enriched in the toll-like receptor (TLR) signaling pathway, non-alcoholic fatty liver disease, and nod-like receptor (NLR) signaling pathways. As the most significant member in NLR family, NLRP3 requires input from both TLR and NLR pathways so as to form inflammasome and mediate the release of IL-1β, IL-18 and HMGB1 [18]. Consistently, the correlation score of IL-1β is greater than 4.0, while the scores of IL-18 and HMGB1 are greater than 1.5, indicating that NLRP3 inflammasome is involved in the development of microvascular hyperpermeability. Thus we fed both *NLRP3*^+*/*+^ and *NLRP3*^*−/−*^ mice with a MCD diet or HFD. As shown in Fig. 2C and D, the expression levels of cleaved-caspase-1 and IL-1β in the coronary microvascular endothelium were markedly increased in NAFLD mice, while NLRP3 gene deficiency significantly blocked these effects. Furthermore, the content of IL-1β and HMGB1 in the cardiac tissue was also reduced in *NLRP3-/-* mice (Additional file 1: Fig. S4A–D). Correspondingly, the disruption of ZO-1/2 integrity and endothelial hyperpermeability in NAFLD mice were prominently recovered in the coronary microvessels by NLRP3 gene knockout (Fig. 2E–H). These results demonstrate that the disruption of endothelial barrier integrity of coronary microvessels is mediated by NLRP3 inflammasome activation during NAFLD.

### Circulating NAFLD hepatic sEV induces NLRP3 inflammasome-dependent endothelial hyperpermeability in coronary microvessels

**Fig. 3:**
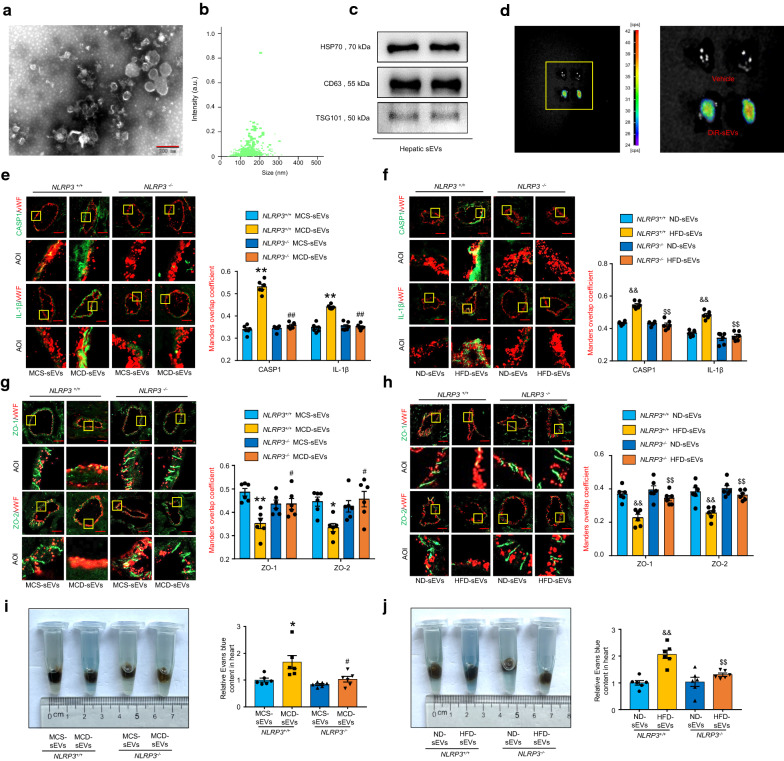
Circulating NAFLD hepatic sEV induces NLRP3 inflammasome-dependent endothelial hyperpermeability in coronary microvessels. NAFLD or control hepatic sEVs were isolated by differential ultracentrifugation, identified, and administered to naive *NLRP3*^+*/*+^ and *NLRP3*^*−/−*^ mice via caudal vein injection. **A** Representative electron micrograph of sEVs reveals the morphology and size. Scale bar, 200 nm. **B** Size distribution analysis of sEVs by nanoparticle tracking analysis. **C** Western blot analyses of sEV markers, including CD63, HSP70, and TSG101. **D** In vivo optical imaging system-obtained fluorescence imaging of cardiac tissue, n = 6 per group. The pellet derived from the ultracentrifugation of DiR alone was used as a vehicle control. **E**–**H** Representative fluorescent confocal images of cleaved-caspase-1(CASP1), IL-1β, and ZO-1/2 (green) with vWF (red) and the summarized data of the Manders overlap coefficient. The area of interest (AOI) is selected for higher magnification, n = 6 per group. Scale bar, 20 μm. **I, J** Representative images and the summarized data of Evans blue concentrations in heart tissues, n = 6 per group. Data are expressed as the mean ± SEM. Statistics: One-way ANOVA, *P < 0.05, **P < 0.01 vs. *NLRP3*^+*/*+^ mice injected with MCS hepatic sEVs; ^#^*P* < 0.05, ^##^*P* < 0.01 vs. *NLRP3*^+*/*+^ mice injected with MCD hepatic sEVs. ^&&^P < 0.01 vs. *NLRP3*^+*/*+^ mice injected with ND hepatic sEVs; ^$$^P < 0.01 vs. *NLRP3*^+*/*+^ mice injected with HFD hepatic sEVs

To explore the role of hepatic sEVs in contributing to microvascular endothelial hyperpermeability, the saucer-shaped, 50–200 nm-sized sEVs were collected and identified using a transmission electron microscopy and a nanosight particle analyzer. Furthermore, the expression of the typical markers including heat shock protein 70 (HSP70), CD63, and tumor susceptibility hum 101 (TSG101) were detected by WB analysis (Fig. 3A–C). The concentration and average size of hepatic sEVs were not changed in both of the two models (Additional file 1: Fig. S9). We then injected DiR-labeled hepatic sEVs into naive mice via the caudal vein. The fluorescence signals were observed in cardiac, pulmonary, hepatic and splenic tissues using an in vivo optical imaging system 12 h later (Fig. 3D, Additional file 1: Fig. S2A–C), suggesting that hepatic sEVs reached the heart, lung, liver and spleen through circulation. Subsequently, sEVs derived from the hepatic tissue (Hepatic sEVs) of NAFLD mice or control mice were injected into *NLRP3*^+*/*+^ and *NLRP3*^*−/−*^ mice. The result showed that both MCD and HFD hepatic sEVs were able to induce the expression of cleaved-caspase-1 and IL-1β in the coronary microvascular endothelium, while NLRP3 gene deficiency markedly reversed these effects (Fig. 3E, F). In addition, NAFLD hepatic sEVs enhanced the cardiac content of HMGB1 and IL-1β in *NLRP3*^+*/*+^ mice but not *NLRP3*^*−/−*^ mice (Additional file 1: Fig. S5A–D). Correspondently, NLRP3 gene knockout restored the ZO-1/2 impairment and microvascular hyperpermeability induced by circulating NAFLD hepatic sEVs (Fig. 3G–J). These in vivo results revealed that hepatic sEVs have a stimulative role in NLRP3 inflammasome-dependent endothelial hyperpermeability in coronary microvessels during NAFLD. In addition, circulating NAFLD hepatic sEVs significantly induced pulmonary microvascular hyperpermeability, but did not change the permeability of hepatic and splenic tissues (Additional file 1: Fig. S2D–F).

### NAFLD hepatic sEVs induce microvascular endothelial hyperpermeability by activating Cathepsin B/NLRP3 inflammasome axis

**Fig. 4:**
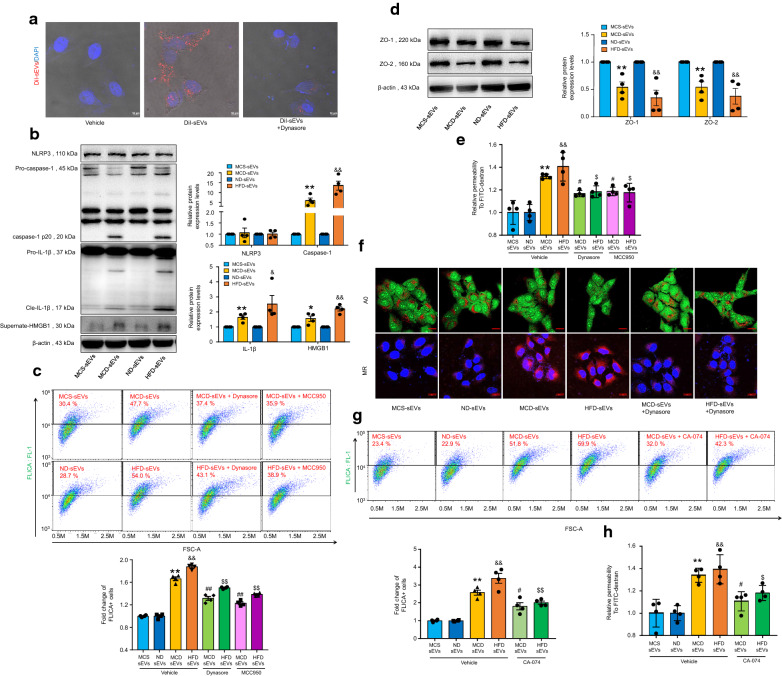
NAFLD hepatic sEVs induce microvascular endothelial hyperpermeability by activating Cathepsin B-dependent NLRP3 inflammasome axis. **A** MVECs were pretreated with or without 20 μmol/L dynasore for 40 min, followed by incubation of 120 μg/mL DiI-labeled hepatic sEVs for 8 h. Representative fluorescent confocal images of DiI-labeled hepatic sEVs (red) with DAPI (blue), n = 4 per group. The pellet derived from the ultracentrifugation of DiI alone was used as a vehicle control. Scale bar, 10 μm. **B** Representative western blot bands and the summarized data determined by densitometric analysis, n = 4 per group. **C** Representative flow cytometry images of caspase-1 FLICA staining and the summarized data of the positive cells, n = 4 per group. The fold changes were obtained by calculating the ratio of the positive cells of the treated groups to the MCS or ND group. **D** Representative Western blot bands and the summarized data determined by densitometric analysis, n = 4 per group. **E** Relative permeability of endothelial monolayer to FITC-dextran, n = 4 per group. **F** Representative images of acridine orange (AO) and magic red (MR) staining, n = 4 per group. Scale bar, 20 μm. **G** MVECs were incubated with 120 μg/mL hepatic sEVs for 24 h with or without the presence of 5 μmol/L CA-074. Representative flow cytometry images of caspase-1 FLICA staining, n = 4 per group. The fold changes were obtained by calculating the ratio of the positive cells of the treated groups to the MCS or ND group. **H** Relative permeability of endothelial monolayer to FITC-dextran, n = 4 per group. Data are expressed as the mean ± SEM. Statistics: Student *t*-test (**B**, **D**) and One-way ANOVA (**C**, **E**, **G**, **H**), *P < 0.05, **P < 0.01 vs. MVECs incubated with MCS hepatic sEVs; ^#^*P* < 0.05, ^##^*P* < 0.01 vs. MVECs incubated with MCD hepatic sEVs; ^&^*P* < 0.05, ^&&^*P* < 0.01 vs. MVECs incubated with ND hepatic sEVs; ^$^*P* < 0.05, ^$$^*P* < 0.01 vs. MVECs incubated with HFD hepatic sEVs

We further confirmed the effects of hepatic sEVs on microvascular endothelial cells through in vitro experiments. We incubated MVECs with DiI-labeled hepatic sEVs for 8 h. Results showed that hepatic sEVs could be internalized by MVECs, while this effect was significantly blocked by dynasore, an endocytosis inhibitor (Fig. 4A). The 3D cell model was constructed from the Z-stack for precise visualization (Additional file 1: Fig. S13). As shown in Fig. 4B, incubation of NAFLD hepatic sEVs promoted the cleavage of caspase-1, maturation of IL-1β, and release of HMGB1, but did not affect NLRP3 protein expression. Activation of NLRP3 inflammasome was further confirmed by the flow cytometry analysis using a caspase-1 FLICA reagent FAM-YVAD-FMK, which was reversed by both dynasore and MCC950, a selective NLRP3 inflammasome inhibitor (Fig. 4C). In addition, NAFLD hepatic sEVs significantly reduced the expression of ZO-1/2 (Fig. 4D) and increased the amounts of FITC-dextran molecules across the endothelial monolayer, which was ameliorated by dynasore and MCC950 (Fig. 4E). We further verified the effect of NLRP3 gene silencing through transfection of NLRP3 siRNA (siNLRP3). As expected, siNLRP3 markedly attenuated caspase-1 activity and improved microvascular endothelial integrity (Additional file 1: Fig. S10).The escape and activation of lysosomal cathepsin B induced by elevated lysosomal membrane permeability (LMP) has been reported as a significant mechanism of NLRP3 inflammasome activation [19]. Thus, we measured the effect of hepatic sEVs on LMP by acridine orange (AO) staining and the cathepsin B activity by magic red (MR) staining. The results showed that NAFLD hepatic sEVs significantly promoted lysosomal permeabilization and induced cathepsin B leakage, which were abolished by dynasore (Fig. 4F). In addition, the presence of CA-074, a specific cathepsin B inhibitor, prominently suppressed NLRP3 inflammasome activation (Fig. 4G) and improved microvascular endothelial hyperpermeability (Fig. 4H). Together, these results imply that NAFLD hepatic sEVs markedly activate Cathepsin B/NLRP3 inflammasome axis, which in turn promote HMGB1 release and induce microvascular endothelial hyperpermeability.

### NAFLD hepatic sEVs-contained novel-miR-7 triggers NLRP3 inflammasome-associated endothelial hyperpermeability

**Fig. 5:**
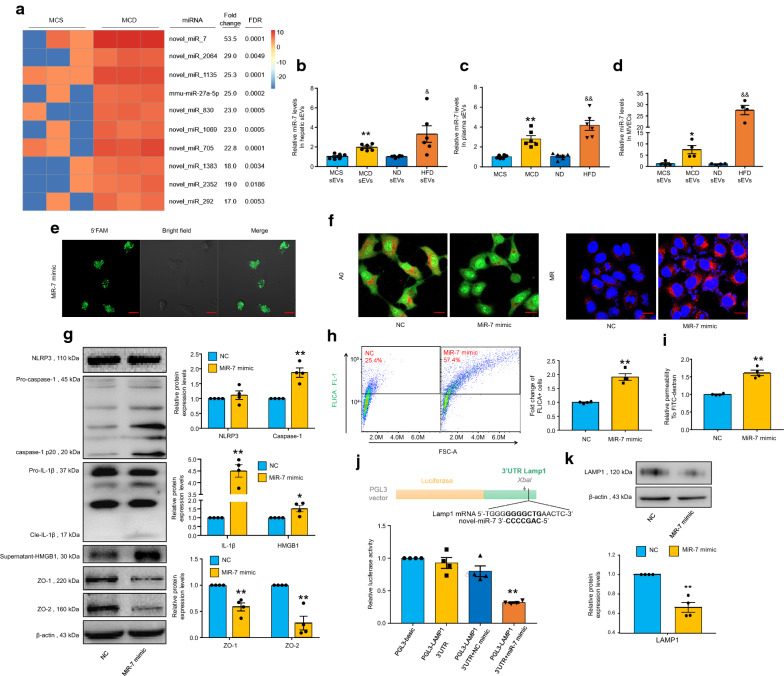
Novel-miR-7 is a key hepatic sEV cargo that promotes microvascular endothelial hyperpermeability by targeting LAMP1. Hepatic sEV miRNAs were profiled by the small RNA sequencing analysis. MVECs were incubated with 120 μg/mL hepatic sEVs for 24 h (**D**) or transfected with 50 nmol/L novel-miR-7 mimics or negative control (NC) mimic (**E**–**K**). **A** The heat-map of the ten miRNAs with the most significant (fold change > 10, FDR < 0.05) abundance differences in hepatic sEVs derived from the MCD group versus the MCS group, n = 3 per group. **B**, **C** Quantitative PCR analysis of novel-miR-7 expression levels in hepatic sEVs and mouse plasma-derived sEVs, n = 6 per group. **D** Quantitative PCR analysis of novel-miR-7 expression levels in hepatic sEVs-treated MVECs, n = 4 per group. **E** Transfection efficiency of 5-FAM labeled novel-miR-7 mimics. Scale bar, 20 μm. **F** Representative images of acridine orange (AO) and magic red (MR) staining, n = 4 per group. Scale bar, 20 μm. **G** Representative Western blot bands and the summarized data determined by densitometric analysis, n = 4 per group. **H** Representative flow cytometry images of caspase-1 FLICA staining and the summarized data of the positive cells, n = 4 per group. The fold changes were obtained by calculating the ratio of the positive cells of the treated groups to the NC group. **I** Relative permeability of the endothelial monolayer to FITC-dextran, n = 4 per group. **J** LAMP1 reporter gene activity was performed using a dual-luciferase reporter assay system and normalized relative to *Renilla* luciferase activity, n = 4 per group. **K** Representative western blot bands and the summarized data determined by densitometric analysis, n = 4 per group. Data are expressed as the mean ± SEM. Statistics: Student *t*-test (**B**–**D**, **G**–**I**, **K**) and One-way ANOVA (**J**), *P < 0.05, **P < 0.01 vs. hepatic sEVs or plasma-sEVs derived from MCS group, or MVEC treated with MCS-sEVs, or NC mimic; ^&^*P* < 0.05, ^&&^*P* < 0.01 vs. hepatic sEVs or plasma-sEVs derived from ND group, or MVEC treated with ND-sEVs

To investigate how NAFLD hepatic sEVs induced NLRP3 inflammasome-associated endothelial hyperpermeability, the miRNA deep sequencing was conducted between hepatic sEVs derived from the MCS and MCD groups. Ten miRNAs with the most significant (fold change > 10, FDR < 0.05) abundance differences are shown in Fig. 5A. Among these differentially expressed miRNAs, novel-miR-7 was the miRNA with the most significant abundance difference. As shown in Fig. 5B and C, novel-miR-7 was significantly elevated in NAFLD hepatic sEVs and plasmatic sEVs. Furthermore, incubation of NAFLD hepatic sEVs led to a prominent abundance of novel-miR-7 in MVECs (Fig. 5D). Subsequently, we investigated the effect of novel-miR-7 on MVECs by transfecting novel-miR-7 mimics. The transfection efficiencies were determined by confocal microscopy, as shown in Fig. 5E. Overexpression of novel-miR-7 significantly promoted lysosomal permeabilization and increased cathepsin B activity (Fig. 5F). The levels of cleavage of caspase-1, maturation of IL-1β, and release of HMGB1 were increased in novel-miR-7-overexpressed cells, while the expression of NLRP3 did not changed (Fig. 5G, H). Correspondently, novel-miR-7 mimics markedly reduced the expression of ZO-1/2 (Fig. 5G) and induced microvascular endothelial hyperpermeability (Fig. 5I). These data indicate that novel-miR-7 could serve as a crucial hepatic sEV cargo. Thus, we performed miRNA target prediction and found that LAMP1 was a target gene of novel-miR-7. LAMP1 was known as a lysosomal membrane glycoprotein and was crucial in regulating LMP [20]. The result of dual-luciferase reporter gene assay showed that co-transfection of novel-miR-7 mimics with the pGL3-LAMP1-3′-UTR plasmid resulted in a significant reduction in fluorescence intensity (Fig. 5J). Furthermore, novel-miR-7 mimics prominently reduced LAMP1 protein expression (Fig. 5K). Together, our results indicate that novel-miR-7 directly targets the 3′-UTR of LAMP1 and induces lysosomal permeabilization, which in turn triggers cathepsin B/NLRP3 inflammasome axis and promotes microvascular endothelial hyperpermeability.

### Genetic inhibition of novel-miR-7 ameliorates NAFLD hepatic sEV-induced microvascular endothelial hyperpermeability

**Fig. 6:**
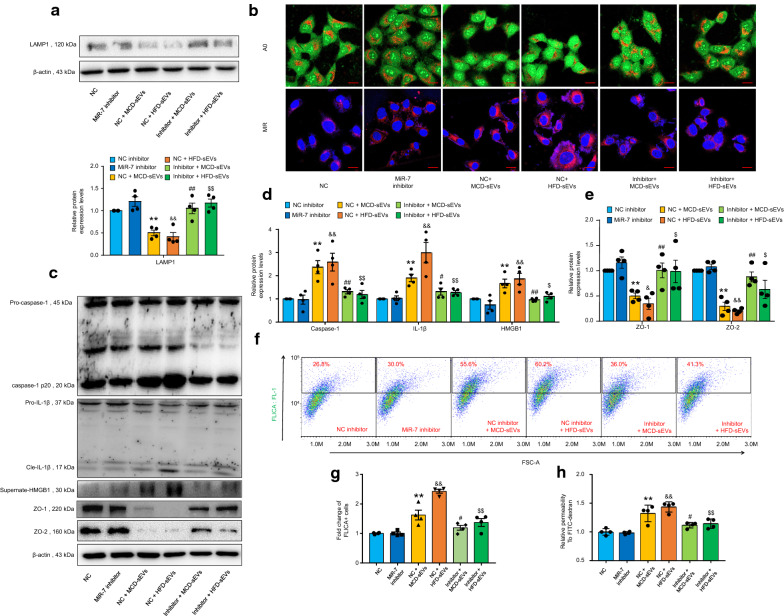
Genetic inhibition of novel-miR-7 ameliorates NAFLD hepatic sEV-induced microvascular endothelial hyperpermeability. MVECs were transfected with 100 nmol/L novel-miR-7 inhibitor or negative control (NC) inhibitor and incubated with or without 120 μg/mL NAFLD hepatic sEVs for 24 h. **A** Representative western blot bands and the summarized data determined by densitometric analysis, n = 4 per group. **B** Representative images of acridine orange (AO) and magic red (MR) staining, n = 4 per group. Scale bar, 20 μm. **C** Representative western blot bands, n = 4 per group. **D** The summarized data of caspase-1, IL-1β and supernate HMGB1 of WB analysis determined by densitometric analysis, n = 4 per group. **E** The summarized data of ZO-1 and ZO-2 of WB analysis determined by densitometric analysis, n = 4 per group. **F** Representative flow cytometry images of caspase-1 FLICA staining, n = 4 per group. **G** The summarized data of the positive cells of FLICA staning, n = 4 per group. The fold changes were obtained by calculating the ratio of the positive cells of the treated groups to the NC inhibitor group. **H** Relative permeability of the endothelial monolayer to FITC-dextran, n = 4 per group. Data are expressed as the mean ± SEM. Statistics: One-way ANOVA, **P < 0.01 vs. NC inhibitor group; ^#^*P* < 0.05, ^##^*P* < 0.01 vs. NC inhibitor plus MCD hepatic sEVs group; ^&^*P* < 0.05, ^&&^*P* < 0.01 vs. NC inhibitor group; ^$^*P* < 0.05, ^$$^*P* < 0.01 vs. NC inhibitor plus HFD hepatic sEVs group

To confirm the significant role of novel-miR-7 in regulating hepatic sEV-induced microvascular endothelial permeability during NAFLD, we transfected MVECs with a specific novel-miR-7 inhibitor (GenePharma, Shanghai, China) and found that novel-miR-7 genetic inhibition markedly recovered LAMP1 deficiency, restored lysosomal permeabilization and ameliorated cathepsin B leakage induced by NAFLD hepatic sEVs (Fig. 6A, B). Consistent with these results, the novel-miR-7 inhibitor prominently suppressed cleavage of caspase-1, maturation of IL-1β and release of HMGB1 (Fig. 6C–G). Furthermore, novel-miR-7 suppression markedly restored the expression of ZO-1/2 (Fig. 6C, E) and improved microvascular endothelial hyperpermeability (Fig. 6H). Taken together, these data demonstrate that genetic inhibition of novel-miR-7 is capable of neutralizing microvascular endothelial hyperpermeability induced by NAFLD hepatic sEVs through regulating the LAMP1/Cathepsin B/NLRP3 inflammasome axis. We also preliminarily verified the therapeutic effect of novel-miR-7 antagomir (GenePharma, Shanghai, China) in vivo. Results showed that novel-miR-7 antagomir could not ameliorate the metabolic disorders and liver injury induced by MCD diet or HFD (Additional file 1: Fig. S6). However, novel-miR-7 antagomir markedly reduced the content of IL-1β and HMGB1 in the cardiac tissues, which suggested the inhibition of NLRP3 inflammasome activity and pro-inflammatory cytokine release (Additional file 1: Fig. S7A–D). Furthermore, novel-miR-7 antagomir significantly attenuated the microvascular hyperpermeability in both heart (Additional file 1: Fig. S7E, F) and lung (Additional file 1: Fig. S8D, E), which indicated that novel-miR-7 may serve as a potential therapeutic target for microvascular complications of NAFLD.

### Steatotic hepatocyte is a significant source of novel-miR-7-enriched sEV

**Fig. 7:**
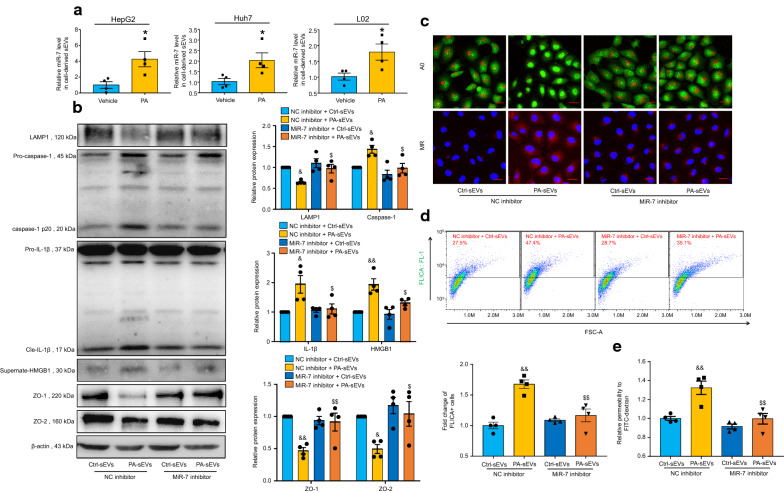
Steatotic hepatocyte is a significant source of novel-miR-7-enriched sEV. Human hepatocyte cell line were treated with 100 μmol/L palmitic acid (PA) or vehicle for 18 h. Hepatocyte sEVs in the HepG2 cell culture media were collected by ultracentrifugation. Human microvascular endothelial cell line-1 (HMEC-1) was transfected with 100 nmol/L novel-miR-7 inhibitor or negative control (NC) inhibitor, and incubated with Ctrl-sEVs or PA-sEVs from HepG2 for 24 h. **A** HepG2, HuH7 and L02 were treated with 100 μmol/L PA or vehicle for 18 h. SEVs were collected from the supernatant and the expression levels of novel-miR-7 were measured by qPCR analysis, n = 4 per group. **B** Representative western blot bands and the summarized data determined by densitometric analysis, n = 4 per group. **C** Representative images of acridine orange (AO) and magic red (MR) staining, n = 4 per group. Scale bar, 20 μm. **D** Representative flow cytometry images of caspase-1 FLICA staining and the summarized data of the positive cells, n = 4 per group. The fold changes were obtained by calculating the ratio of the positive cells of the treated groups to the NC inhibitor group. **E** Relative permeability of the endothelial monolayer to FITC-dextran, n = 4 per group. Data are expressed as the mean ± SEM. Statistics: Student t-test (**A**) and One-way ANOVA (**B**, **D**, **E**), *P < 0.05, **P < 0.01 vs. vehicle group; ^&^*P* < 0.05, ^&&^*P* < 0.01 vs. NC inhibitor plus ctrl-sEVs group; ^$^*P* < 0.05, ^$$^*P* < 0.01 vs. NC inhibitor plus PA-sEVs

To explore the origin of novel-miR-7-contained hepatic sEVs, we treated human hepatocyte cell line HepG2, Huh7 and L02 with 100 μmol/L palmitic acid (PA) for 18 h, which induced the accumulation of intracellular lipid but did not cause cell death (Additional file 1: Fig. S3, A–D). Result showed that novel-miR-7 was significantly abundant in steatotic hepatocyte-derived sEVs (Fig. 7A). We then transfected human microvascular endothelial cell line-1 (HMEC-1) with novel-miR-7 inhibitor or NC inhibitor and stimulated with HepG2-derived sEVs for 24 h. Results showed that PA-sEVs triggered LAMP1/Cathepsin B/NLRP3 inflammasome axis, which was markedly abolished by novel-miR-7 inhibitor (Fig. 7B–D). Furthermore, the novel-miR-7 inhibitor restored the tight junction disruption and microvascular endothelial hyperpermeability induced by PA-sEVs (Fig. 7B, E). These results indicate that steatotic hepatocyte is a significant source of novel-miR-7-contained hepatic sEVs, and steatotic hepatocyte-derived sEVs are able to promote NLRP3 inflammasome-dependent microvascular endothelial hyperpermeability through novel-miR-7.

## Discussion

This study aims to investigate the role of hepatic sEVs in regulating microvascular endothelial hyperpermeability during NAFLD. This will broaden the understanding of the liver-to-microvessel cross-talk, which may serve as a significant pathogenic mechanism contributing to the microvascular complications of NAFLD.

Multiple studies have proved that CMD is associated with increased risk of major cardiovascular events [21]. In this study, we found that NAFLD induces endothelial barrier dysfunction and hyperpermeability in coronary microvessels. In recent years, several studies have provided evidence that extracellular HMGB1 contributes to endothelial tight junction disruption and hyperpermeability through the receptor for advanced glycation end products-mediated signaling pathway in chronic inflammation [12, 22]. Enhanced HMGB1 release could be induced by activated NLRP3 inflammasomes and participate in inflammatory responses [23]. In line with previous studies, we found by searching GeneCards dataset that the activities of NLRP3 inflammasome-mediated cytokines including IL-1β, IL-18 and HMGB1 are the human targets of microvascular hyperpermeability. We subsequently demonstrated that the NLRP3 inflammasome is activated in coronary microvascular endothelium during NAFLD and the release of cytokines including IL-1β and HMGB1 are elevated in cardiac tissue. NLRP3 gene knockout conspicuously blocked these effects and relieved endothelial hyperpermeability, demonstrated that endothelial hyperpermeability in coronary microvessels depends on NLRP3 inflammasome activation during NAFLD.

In the present study, we used both a MCD diet and HFD to induce intrahepatic lipid accumulation. Mice fed a HFD is a commonly-used model of NAFLD, which could develop the metabolic syndrome and hepatic steatosis. Compared to HFD, mice fed a MCD diet do not reproduce some crucial metabolic features of human NAFLD including obesity, hyperlipemia and insulin resistance [24]. It has been previously proved that NLRP3 inflammasome-mediated HMGB1 release plays a vital role in endothelial hyperpermeability during several metabolic disorders such as obesity, hyperglycemia, and diabetes [11, 25, 27]. However, microvascular endothelial barrier dysfunction and hyperpermeability were observed in NAFLD mice induced by both MCD and HFD, which indicated that these effects do not only rely on the metabolic features induced by NAFLD but also an independent pathogenic factor. This brought us to investigate the mediators of the liver-to-microvessels communication pathway.

In recent years, hepatic sEVs have emerged as crucial substances in intercellular and inter-organ communication during NAFLD [28, 28]. For example, hepatocyte sEVs derived from NAFLD mice facilitate macrophage M1 polarization and activation [29]. A recent study showed that steatotic hepatocyte-derived sEVs contribute to endothelial inflammation and atherogenesis [17]. These studies suggested a crucial pro-inflammatory role of hepatic sEVs during NAFLD. Thus, we assumed that NAFLD induced microvascular endothelial hyperpermeability through the release of hepatic sEVs. We then labeled hepatic sEVs with DiR and proved that DiR-sEVs reached the cardiac tissue through circulation. Through in vitro experiment, we proved that DiI-hepatic sEVs could be internalized by microvascular endothelial cells via endocytosis. We subsequently found that sEVs derived from steatotic liver promote NLRP3 inflammasome-dependent microvascular endothelial hyperpermeability, suggested that hepatic sEVs might be an independent pathogenic factor of CMD independent from the metabolic disorders during NAFLD. In addition, we investigated the stimulative effect of hepatic sEVs on other main visceral organs. A previous study has reported that circulating sEVs were taken up by macrophages in the liver and spleen and by endothelial cells in the lung [30]. In line with this study, we found that circulating hepatic sEVs reached pulmonary, hepatic and splenic tissues and promoted pulmonary microvascular hyperpermeability, but did not affect the hepatic and splenic tissues. These findings indicate that the recipient cells of circulating hepatic sEVs varies in different organs.

Among the biological substances in sEV cargos, non-coding RNAs, especially miRNAs, have flourished in the past few years [31]. Recent studies have revealed the biological effects of hepatic sEV miRNAs during NAFLD. For instance, miR-192-5p derived from hepatocyte sEVs is crucial in the activation of proinflammatory macrophages and participates in NAFLD progression by regulating Rictor/Akt/FoxO1 signaling [29]. MiR-1 derived from steatotic hepatocyte sEVs promotes endothelial inflammation and atherogenesis by reducing KLF4 expression [17]. MiRNA let-7e-5p derived from hepatocyte sEVs contributes to adipose remodelling by targeting Pgc1α [32]. In this study, we identified a novel miRNA, novel-miR-7, as an important bioactive hepatic sEV cargo. Novel-miR-7 was the most significantly expressed miRNA in hepatic sEVs. It is believed that relatively high-expressing miRNA possesses prominent biological effects. According to our results, novel-miR-7 were elevated in NAFLD hepatic and plasmatic sEVs, indicating that novel-miR-7 could be released into the circulation as a hepatic sEV cargo. We then demonstrated that novel-miR-7 was abundant in MVECs after incubation of NAFLD hepatic sEVs, while overexpression of novel-miR-7 promoted microvascular endothelial barrier function through NLRP3 inflammasome activation. Moreover, novel-miR-7 genetic inhibition significantly restored NLRP3 inflammasome-dependent microvascular endothelial hyperpermeability induced by NAFLD hepatic sEVs both in vivo and in vitro. Furthermore, we provided preliminary evidences that the mechanism of pulmonary microvascular hyperpermeability is mediated by novel-miR-7, which is similar to coronary microvessels (Additional file 1: Fig. S8). Taken together, these results implied that novel-miR-7 may act as a diagnostic biomarker and therapeutic target for NAFLD-associated microvascular complications. It should be noted that genetic inhibition of novel-miR-705 and novel-miR-1135, which are the significant changed miRNAs with relatively higher expression (Fig. 5A, Additional file 3: Table S2), did not show conspicuous effect on inhibiting NLRP3 inflammasome (Additional file 1: Fig. S11), thus we did not conduct deeper studies on the function of novel-miR-705 and novel-miR-1135.

To date, several molecular mechanisms that mediate NLRP3 inflammasome activation have been widely investigated, including K^+^ efflux, elevated reactive oxygen species production and increased cathepsin B activity [33]. In this study, we demonstrated that NLRP3 inflammasome activation induced by novel-miR-7-contained hepatic sEVs depends on lysosomal permeabilization and enhanced cathepsin B activity. LAMP1 is a predominant lysosomal membrane protein that maintains lysosomal membrane integrity, pH, and catabolism [34]. The reduction of LAMP1 is associated with decreased lysosome integrity and elevated cathepsin B leakage [35, 36]. In the present study, we performed dual-luciferase reporter assay and demonstrated that novel-miR-7 directly bound with the 3′-UTR of LAMP1 mRNA and inhibited LAMP1 transcription. Furthermore, the specific novel-miR-7 inhibitor remarkably restored LAMP1 expression, lysosomal permeabilization and Cathepsin B leakage, followed by improvement of NLRP3 inflammasome-dependent microvascular endothelial hyperpermeability. These findings suggest that the anti-novel-miR-7 sequence has the therapeutic potential for CMD during NAFLD by regulating LAMP1/Cathepsin B/NLRP3 inflammasome axis.

In the present study study, we attempted to explore the origin of novel-miR-7. Hepatocytes are known as the main kind of cells in the liver and primarily constitute the liver parenchyma. EVs released from impaired hepatocytes contribute to the development of inflammation and fibrosis [37]. Here, we found that steatotic hepatocytes are significant sources of novel-miR-7 enriched hepatic sEVs, and genetic inhibition of novel-miR-7 markedly improved microvascular hyperpermeability (Fig. 7). However, one of the limitation of this study is that we did not measure the novel-miR-7 expression in sEVs derived from non-parenchymal cells such as hepatic stellate cells (HSCs) and Kupffer cells, which may also serve as significant donors of pathogenetic sEVs. For instance, EVs derived from activated HSCs could enhance or fine tuning of fibrogenic signaling through transferring the connective tissue growth factor [38]. Hence the roles of non-parenchymal cells-derived sEVs in the development of microvascular complications during NAFLD remain to be further investigated.

## Conclusions

In this study, we provide compelling evidence that endothelial hyperpermeability occurs in coronary microvessels during NAFLD. Enhanced intrahepatic lipids level induces the secretion of novel-miR-7-abundant hepatic sEVs and promotes microvascular endothelial hyperpermeability by regulating the LAMP1/Cathepsin B/NLRP3 inflammasome axis. Furthermore, steatotic hepatocyte is a significant source of novel-miR-7-contained sEVs, and genetic inhibition of novel-miR-7 improves microvascular endothelial barrier integrity. Thus, our study brings new insights into the inter-organ communication mechanisms between the liver and coronary microvessels, and may provide a new diagnostic biomarker and therapeutic target for the microvascular complications of NAFLD.

## Methods

### Animal experiments

All animal studies followed the guidelines of the National Institutes of Health (NIH) and were approved by the Institutional Animal Care and Use Committee of Guangzhou University of Chinese Medicine. Male wild-type (*NLRP3*^+*/*+^) C57BL/6 N mice (8–10 weeks) were purchased from Vital River Laboratory (Beijing, China). *NLPR3*^*L351PneoR*^ mice (*NLRP3*^−/−^) were purchased from Jackson Laboratory (Sacramento, CA, USA), bred, and reproduced. *NLRP3*^+*/*+^ and *NLRP3*^−/−^ mice were genotyped following the protocol by the vendor. Mice were housed in a temperature-controlled facility with a 12-h light/dark cycle and were given free access to food and water. In the first NAFLD model, mice were fed a methionine-choline-deficient (MCD) diet (Trophic Animal Feed High-tech Co., Nantong, China) or a methionine-choline-supplemented (MCS) diet for 4 weeks. In the second NAFLD model, mice were fed a 60 kcal% high-fat diet (HFD) (Research Diets Inc., Brogaarden, Denmark) or normal diet (ND) for 16 weeks. For the in vivo hepatic sEV function experiments, mice were injected with 20 mg/kg hepatic sEVs via the caudal vein for 2 days. For euthanasia, mice were anaesthetized by 3 vol% isoflurane and sacrificed by exsanguination. For the microRNA antagomir experiments, after being fed with a MCD diet for 2 weeks or a HFD for 6 weeks, mice were injected with 1 μmol/kg novel-miR-7 antagomir or negative control antagomir via the caudal vein once a week. The sequences of novel-miR-7 antagomir and negative control antagomir were listed in Additional file 2: Table S1.

### Cell cultures and treatments

Mouse microvascular endothelial cell (MVEC) line EOMA, human microvascular endothelial cell line-1 (HMEC-1), mouse lung microvascular endothelial cell line (MLVEC), and human hepatocyte cell line L02 were obtained from BeNa Culture Collection (Beijing, China). Human hepatocyte cell line HepG2 were obtained from ATCC (Manassas, VA, USA). Human hepatocyte cell line Huh7 was obtained from the Japanese Collection of Research Bioresources Cell Bank (Osaka, Japan). HMEC-1 was maintained in Endothelial Culture Media (ScienCell, Carlsbad, CA, USA), L02 was maintained in RPMI 1640 culture media (Gibco, Carlsbad, CA, USA), and other cell lines were maintained in Dulbecco’s modified eagle medium (Gibco, Carlsbad, CA, USA) in a humidified incubator with 5% CO_2_ at 37 °C. Dynasore, MCC950, and CA-074 were purchased from MedChemExpress (Monmouth Junction, NJ, USA) and dissolved in DMSO. Palmitic acid (PA) was dissolved in 0.1 mol/L NaOH by heating at 75 °C for 30 min and conjugated with fatty acid-free BSA solution. Cells were incubated with 120 μg/mL hepatic sEVs in EV-free FBS-supplemented media (SBI System Biosciences, Palo Alto, CA, USA) for 24 h.

### Histology

Liver paraffin sections (4-μm-thick) were stained with hematoxylin and eosin (Beyotime Biotechnology, Shanghai, China) according to the manufacturer’s protocol. Frozen liver sections (8-μm-thick) were stained with Oil red O (Biotopped, Beijing, China) according to the manufacturer’s instructions. Sections were observed and photographed at 400 × magnification using a CX31 microscope (Olympus, Tokyo, Japan).

### Doppler ultrasound imaging

Doppler ultrasound studies were performed using a Vevo 2100 Imaging System (VisualSonics, Toronto, Canada) with a MS400 linear-array transducer. Liver echogenicity were evaluated in the B-mode.

### Measurement of aminotransferase activity and lipid content

The aminotransferase activity (aspartate aminotransferase and alanine aminotransferase) and lipid content (total cholesterol, triglyceride and low-density lipoprotein cholesterol) in plasma samples, liver samples or hepatocytes were determined using commercial kits according to the manufacturer’s protocol (Nanjing Jiancheng Biology, Nanjing, China).

### ELISA analysis

The contents of HMGB1 and IL-1β in the heart tissue were measured by commercial ELISA kits (Meimian Industrial, Wuhan, China) following the manufacturer’s instructions. Protein concentration was determined using bicinchoninic acid protein assay kits (Beyotime, Beijing, China). The results of the cytokine concentration were normalized to protein concentration.

### Immunofluorescence analysis

For tissue immunofluorescence staining, frozen cardiac Sects. (8-μm-thick) were fixed in pre-cooled acetone or 4% paraformaldehyde and blocked with 10% donkey serum for 1 h at room temperature, then incubated overnight at 4 °C for 1 h at room temperature with the primary antibodies. After washing with PBS, the slides were incubated with the corresponding Alexa Fluor 488 or Alexa Fluor 555-labeled secondary antibodies for 2 h at room temperature. The sections were visualized using a LSM 800 laser confocal scanning microscope (Carl Zeiss, Oberkochen, Germany). The Manders overlap coefficient was obtained by using the Image Pro-Plus 6.0 software and used in quantitative image analysis. Anti-cleaved-caspase-1 primary antibodies were purchased from Santa Cruz Biotechnology (Santa Cruz, CA, USA). Anti-IL-1β and vWF primary antibodies were purchased from Abcam (Cambridge, MA, USA). Anti-ZO-1/2 primary antibodies and fluorescent secondary antibodies were purchased from Invitrogen (Carlsbad, CA, USA).

### Microvascular endothelial permeability assay

The microvascular endothelial permeability assay was performed as previously described [6]. Briefly, mice were injected with 80 mg/kg Evans blue solution (Sigma-Aldrich, St. Louis, MO, USA) via the caudal vein. Two hours later, mice were sacrificed and injected with PBS through the left ventricle to remove intravascular Evans blue solution. Tissues were collected and incubated in formamide at 60 °C for 2 days. The Evans blue content was quantitated by spectrophotometry at 620 nm and normalized to tissue weight.

### Screening of human disease target and GO enrichment analysis

The GeneCards database (https://www.genecards.org/) was used to gather the information of human targets related to microvascular hyperpermeability. The keyword ‘microvascular hyperpermeability’ was used to screen with a correlation score of ≥ 1.5 as candidate target genes. KEGG pathway enrichment analysis was performed using the clusterProfiler R package. The clusterProfiler R package was used to perform Gene Ontology (GO) enrichment analysis and to visualize the possible biological processes (BP), cellular component (CC) and molecular function (MF) that were involved [39].

### Purification and characterization of sEV

Mouse liver tissues were minced in PBS and the hepatic sEVs were collected according to method of Loyer et al. [40] with minor modifications. HepG2 were treated with 100 μmol/L palmitic acid (PA) or vehicles in EV-free FBS-supplemented media for 18 h, and hepatocyte sEVs were collected from the cell culture media. Briefly, liver samples or cell culture media were centrifuged at 2000×*g* for 30 min at 4 °C to remove dead cells, and 12,000×*g* for 30 min for twice to remove cell debris and large extracellular vesicles. Supernatants were then collected and filtered through 0.22 μm microporous membranes, followed by ultracentrifugation at 110,000 × g for 70 min at 4 °C twice. Finally, the sEV sediment was resuspended in PBS and filtered through 0.22 μm microporous membranes. Protein concentration was determined using bicinchoninic acid protein assay kits (Beyotime, Beijing, China). The sEV particle sizes were determined using nanoparticle tracking analysis with a Nanosight NS 300 (Malvern, Malvern, UK), and the morphology of sEV was identified using a JEM-1200EX transmission electronic microscope (JEOL, Tokyo, Japan).

### In vivo and in vitro sEV internalization assay

Hepatic sEVs were labelled with 10 μmol/L DiR (US Everbright Inc., Suzhou, China) for 30 min at 37 °C or 5 μmol/L DiI (Beyotime, Beijing, China) for 20 min at room temperature. Subsequently, sEVs were collected by ultracentrifugation at 110,000×*g* for 70 min at 4 °C. The DiR or DiI-labeled sEV sediments were resuspended in PBS and filtered through 0.22 μm microporous membranes. The pellets derived from the ultracentrifugation of DiR or DiI alone was used as vehicle controls. For the in vivo internalization assay, hepatic sEVs were administrated to naive mouse via caudal vein injection. Twelve hours later, tissues were harvested and imaged by LB983 in vivo optical imaging system (Berthold, Bad Wildbad, Germany). For the in vitro internalization assay, MVECs were pretreated with or without 20 μmol/L dynasore for 40 min and incubated with DiI-labeled hepatic sEVs. Eight hours later, cells were harvested, fixed with 4% paraformaldehyde, counterstained with DAPI and visualized using a LSM 800 laser confocal scanning microscope (Carl Zeiss, Oberkochen, Germany). For the establishment of 3D cell model, cells were further stained with wheat germ agglutinin (WGA) as a plasma membrane marker and visualized using a stimulated emission depletion (STED) microscopy (Abberior, Göttingen, Germany). The cell 3D model was constructed by using Figi.

### Western blotting

The Western blotting assay was performed as previously described [41]. Briefly, hepatic sEVs, cell lysates or supernatants were boiled at 100 °C for 5 min, separated on 8–15% SDS–polyacrylamide gel and transferred to a 0.22 μm PVDF membrane. Membranes were then blocked with 5% non-fat milk for 1 h at room temperature and incubated with the primary antibodies overnight at 4 °C, followed by incubation with the corresponding horseradish peroxidase-conjugated secondary antibodies. The protein bands were detected using a chemiluminescence imaging system (Tanon, Shanghai, China) and analyzed using Image J software. Densitometric analysis was used to determine the relative protein expression. The bands of caspase-1 p20 were normalized to that of pro-caspase-1. The bands of cleaved-IL-1β were normalized to that of pro-IL-1β. The cell culture supernatant HMGB1 were collected from the same numbers of cells and the bands were normalized to that of cellular GAPDH or β-actin. The rest of bands were normalized to that of GAPDH or β-actin. Anti-caspase-1 primary antibody was purchased from Wanlei Biology (Shenyang, China). Anti-HMGB1, LAMP1, and HSP70 primary antibodies were purchased from Santa Cruz Biotechnology (Santa Cruz, CA, USA). Anti-ZO-1/2 primary antibodies were purchased from Invitrogen (Carlsbad, CA, USA). Anti-IL-1β, NLRP3, TSG101, and CD63 primary antibodies were purchased from Abcam (Cambridge, MA, USA). Anti-β-actin and GAPDH primary antibodies were purchased from Affinity Biologicals (Ancaster, ON, Canada). Anti-mouse and rabbit secondary antibodies were purchased from Cell Signaling Technology (Danvers, MA, USA).

### Analysis of caspase-1 activity

The caspase-1 activity in cells was measured using flow cytometry in strict accordance with the instructions of the FAM-FLICA Caspase Assay Kit (ImmunoChemistry Technologies, Bloomington, MN, USA). Briefly, cells were stained with FAM-YVAD-FMK for 1 h, collected and detected by an Accuri C6 flow cytometer (BD Biosciences, San Jose, CA, USA). Results were analyzed using FlowJo 10.0 Software. The untreated cells were used as negative controls to set a gate for analyzing FLICA-positive cells. The fold changes were obtained by calculating the ratio of the positive cells of the treated groups to the control group.

### Measurement of microvascular endothelial permeability

Endothelial barrier function was assessed by measuring the permeability of MVECs and HMEC-1 to fluorescein isothiocyanate-conjugated dextran (FITC-dextran) as described previously [42]. Briefly, microvascular endothelial cells were seeded on transwell chambers and incubated with hepatic sEVs or hepatocyte sEVs. Twenty-four hours later, the culture medium was replaced with PBS in the lower chamber and 1 mg/mL FITC–dextran 4 kDa (Sigma-Aldrich, St. Louis, MO, USA) in the upper chamber. One hour later, samples from the upper and lower compartments were measured by a fluorescent plate reader using excitation and emission wavelengths of 485 and 535 nm, respectively.

### Analysis of lysosome membrane permeability

Lysosome membrane permeability was determined by acridine orange (AO) staining. The cells were then incubated with AO (Solarbio, Beijing, China) for 15 min at 37 °C according to the manufacturer's guidelines. Live cells were immediately visualized using a LSM 800 laser confocal scanning microscope (Carl Zeiss, Oberkochen, Germany).

### Analysis of cathepsin B activity

The activity of cathepsin B from disrupted lysosomes was determined using the Magic Red (MR) Cathepsin B Assay Kit (ImmunoChemistry Technologies, Bloomington, MN, USA). Briefly, cells were stained with a fluorescent cell-permeable selective cathepsin B substrate MR-(RR)_2_ for 1 h, fixed with 4% paraformaldehyde, counterstained with DAPI, and visualized using a LSM 800 laser confocal scanning microscope (Carl Zeiss, Oberkochen, Germany).

### MiRNA deep-sequencing

Hepatic sEV miRNAs were profiled by the small RNA sequencing analysis in a v3 flowcell on an Illumina HiSeq 2500 sequencer (Illumina Inc., San Diego, CA, USA). Differential expression analysis of the MCS and the MCD groups was performed using the DESeq R package. Prediction of miRNA targets and identification of miRNA binding sites were performed using RNA22 (version 2.0).

### MiRNA real-time quantitative PCR

For the measurement of novel-miR-7 level, plasmatic sEVs were collected using an ExoQuick reagent (SBI System Biosciences, Palo Alto, CA, USA), and hepatocyte sEVs were collected from the cell culture media using a Ribo Exosome Isolation Reagent (Ribobio, Guangzhou, China). Total RNAs in sEVs or MVECs were isolated using TRIzol reagent and reverse transcribed using a miRNA first-strand cDNA synthesis kit (Sangon, Shanghai, China) according to the manufacturer’s guidelines. RT-qPCR was performed using a TB Green™ Premix Ex Taq™ (Takara, Kyoto, Japan) according to the manufacturer’s guidelines. Relative miRNA expression levels were calculated using the comparative threshold (CT) method. Results were normalized to U6 in MVECs or total RNA in sEVs. The primers were purchased from Sangon Biotech, and the sequences are listed in Additional file 2: Table S1.

### Transfection of microRNA mimic, microRNA inhibitor and siRNA

For transfection of the novel-miR-7 mimic, cells were transfected with either a 5-FAM-labelled novel-miR-7 mimic or a mimic negative control (GenePharma, Shanghai, China) at a final concentration of 50 nM using Lipofectamine 3000 (Invitrogen, Carlsbad, CA, USA) according to the manufacturer’s instructions. Transfection efficiencies were determined by confocal microscopy. For transfection of the microRNA inhibitor, including the novel-miR-7, novel-miR-705 and novel-miR-1135 inhibitor, cells were transfected with either a miRNA inhibitor or inhibitor-negative control at a final concentration of 100 nM using Lipofectamine 3000 according to the manufacturer’s instructions. For transfection of siNLRP3, cells were transfected with either a NLRP3 siRNA or negative control siRNA at a final concentration of 100 nM using Lipofectamine 3000 according to the manufacturer's instructions. The sequences are listed in Additional file 2: Table S1.

### Dual-luciferase reporter assay

The 3′-UTR fragment of LAMP1 was generated by PCR and cloned into pGL3 Luciferase Reporter Vector (Promega, Madison, WI, USA), which contained the putative novel-miR-7 binding sequence. pRL-TK vector was co-transfected in each experiment as an internal control. Luciferase activity assays were performed using a dual-luciferase reporter assay system (Promega) and normalized relative to *Renilla* luciferase activity according to the manufacturer’s instructions. The sequence of pGL3-LAMP1-3’UTR plasmid is listed in Additional file 2: Table S1.

### Measurement of cell viability and LDH leakage

Cell viability was assessed by CCK-8 assays according to the manufacturer's protocol. The leakage of lactate dehydrogenase (LDH) was assessed using a LDH assay kit (Nanjing Jiancheng Biology, Nanjing, China) according to the manufacturer's protocol.

### Measurement of lipid accumulation in hepatocytes

Hepatocytes were fixed with 4% paraformaldehyde and stained with oil red o (Biotopped, Beijing, China) for 30 min. Subsequently, the oil red o was extracted by isopropanol and quantitated by spectrophotometry at 510 nm. Cellular triglyceride content was measured according to the manufacturer's protocol (Nanjing Jiancheng Biology, Nanjing, China).

### Statistical analysis

Data are presented as the mean with the standard error of the mean (SEM). Significant differences were examined using unpaired Student *t* test or one-way ANOVA. Statistical analysis was performed using SPSS software. *P* < 0.05 was considered statistically significant.

## Supplementary Information

**Additional file 1: Figure S1.** Body weight, liver weight and circulating lipid content in MCD and HFD-induced NAFLD mice. Mice were fed a MCD diet for 4 weeks or HFD for 16 weeks to induce NAFLD. (A) Body weight of NAFLD mice, n = 6 per group. (B) Representative images of liver and the liver weight of NAFLD mice, n = 6 per group. Scale bar:5 mm. (C-E) Relative plasma total cholesterol (TC), triglyceride (TG) and low-density lipoprotein cholesterol (LDL-C) content, n = 6 per group. Data are expressed as the mean ± SEM. Statistics: Student t-test, *P < 0.05, **P < 0.01 vs. the MCS group; &&P < 0.01 vs. ND group. **Figure S2**. Changes of microvascular endothelial permeability in lung, liver and spleen induced by hepatic sEVs. NAFLD or control hepatic sEVs were isolated by ultracentrifugation, identified, and administered to naive NLRP3+/+ and NLRP3-/- mice via caudal vein injection. (A-C) In vivo optical imaging system-obtained fluorescence imaging of lung, liver and spleen, n = 6 per group. The pellet derived from the ultracentrifugation of DiR alone was used as a vehicle control. (D-F) Representative images and the summarized data of Evans blue concentrations in lung, liver and spleen, n = 5-6 per group. Data are expressed as the mean ±SEM. Statistics: One-way ANOVA, *P < 0.05 vs. NLRP3+/+ mice injected with MCS hepatic sEVs; &&P < 0.01 vs. NLRP3+/+ mice injected with ND hepatic sEVs; ## P < 0.01 vs. NLRP3+/+ mice injected with MCD hepatic sEVs; $$ P < 0.01 vs. NLRP3+/+ mice injected with HFD hepatic sEVs. **Figure S3**. Cell viability, LDH leakage and lipid accumulation in steatotic hepatocytes. Human hepatocyte cell line HepG2, HuH7 and L02 were treated with 100 μmol/L palmitic acid (PA) or vehicle for 18 h. (A) Cell viability were measured using cell counting kit (CCK)-8, n = 6 per group. (B) Lactic dehydrogenase (LDH) leakage was estimated by measuring LDH activities in the cell culture supernatants, n = 6 per group. (C) Intracellular triglyceride (TG) contents were measured by chromometry, n = 4 per group. (D) Hepatocytes were stained with Oil Red O and the intracellular lipid accumulation was measured by chromometry, n = 4 per group. Data are expressed as the mean ±SEM. Statistics: Student t-test, Ns, no significance, **P < 0.01 vs. vehicle group. **Figure S4**. Cytokine contents in the cardiac tissue of NAFLD mice.NLRP3+/+ and NLRP3-/- Mice were fed a MCD diet for 4 weeks or HFD for 16 weeks to induce NAFLD. The contents of cytokines including IL-1β and HMGB1 in the cardiac tissues were measured by ELISA kits. The results were normalized to protein concentration. (A-B) Cardiac IL-1β and HMGB1 content in NLRP3+/+ and NLRP3-/- mice fed a MCS or MCD diet, n = 5-6 per group. (C-D) Cardiac IL-1β and HMGB1 content in NLRP3+/+ and NLRP3-/- Mice fed a ND or HFD, n = 5-6 per group. Data are expressed as the mean ±SEM. Statistics: One-way ANOVA, **P < 0.01 vs. NLRP3+/+ mice fed with a HFD diet. **Figure S5.** Cytokine contents in the cardiac tissue of hepatic. sEV-treated mice NAFLD or control hepatic sEVs were isolated by ultracentrifugation, identified, and administered to naive NLRP3+/+ and NLRP3-/- mice via caudal vein injection. The contents of cytokines including IL-1β and HMGB1 in the cardiac tissues were measured by ELISA kits. The results were normalized to protein concentration. (A-B) Cardiac IL-1β and HMGB1 content in NLRP3+/+ and NLRP3-/- mice injected with MCS-sEVs or MCD-sEVs, n = 5-6 per group. (C-D) Cardiac IL-1β and HMGB1 content in NLRP3+/+ and NLRP3-/- mice injected with ND-sEVs or HFD-sEVs, n = 5-6 per group. Data are expressed as the mean ±SEM. Statistics: One-way ANOVA, **P < 0.01 vs. NLRP3+/+ mice injected with MCS-sEVs; ##P < 0.01 vs. NLRP3+/+ mice injected with MCD-sEVs; &&P < 0.01 vs. NLRP3+/+ mice injected with ND-sEVs; $$P < 0.01 vs. NLRP3+/+ mice injected with HFD-sEVs. **Figure S6.** Novel-miR-7 antagomir does not alter the changes of body weight, circulating lipid content and aminotransferase activities of NAFLD mice. Mice were fed with a MCD diet for 2 weeks or a HFD for 6 weeks, followed by administration of 1 μmol/kg novel miR-7 antagomir or negative control (NC) lipoprotein cholesterol (LDL-C) content, n = 6 per group. (E-F) Plasma levels of aspartate aminotransferase (AST) and alanine aminotransferase (ALT), n = 6 per group. Data are expressed as the mean ± SEM. Statistics: One-way ANOVA, **P < 0.01 vs. the MCS plus NC antagomir group; &P < 0.05, &&P < 0.01 vs. the ND plus NC antagomir group; Ns: no significance, the MCD plus novel-miR-7 antagomir group were compared with the MCD plus NC antagomir group, and the HFD plus novel-miR-7 antagomir group were compared with the HFD plus NC antagomir group. Figure S7 Novel-miR-7 antagomir reduces the cardiac cytokine contents and ameliorates coronary microvascular hyperpermeability of NAFLD mice. Mice were fed with a MCD diet for 2 weeks or a HFD for 6 weeks, followed by administration of 1 μmol/kg novel miR-7 antagomir or negative control (NC) antagomir via the caudal vein once a week. (A-D) Cardiac IL-1β and HMGB1 contents were measured by ELISA kits, n = 5-6 per group. (E-F) Representative images and the summarized data of Evans blue concentrations in heart, n = 6 per group. Data are expressed as the mean ± SEM. Statistics: One-way ANOVA, **P < 0.01 vs. the MCS plus NC antagomir group; &P < 0.05, &&P < 0.01 vs. the ND plus NC antagomir group; #P < 0.05, ##P < 0.01 vs. the MCD plus NC antagomir group; $$P **Figure S8.** NAFLD hepatic sEVs promote pulmonary microvascular hyperpermeability via novel-miR-7. Mouse lung microvascular endothelial cells (MLVECs) were transfected with 100 nmol/L novel-miR-7 inhibitor or negative control (NC) inhibitor and incubated with or without 120 μg/mL NAFLD hepatic sEVs for 24 h. Mice were fed with a MCD diet for 2 weeks or a HFD for 6 weeks, followed by administration of 1 μmol/kg novel miR-7 antagomir or negative control (NC) antagomir via the caudal vein once a week. (A-B) Representative flow cytometry images of caspase-1 FLICA staining and the summarized data of the positive cells, n = 4 per group. The fold changes were obtained by calculating the ratio of the positive cells of the treated groups to the NC inhibitor group. (C) Relative permeability of the endothelial monolayer to FITC-dextran, n = 4 per group. (D-E) Representative images and the summarized data of Evans blue concentrations in lung, n = 6 per group. Data are expressed as the mean ± SEM. Statistics: One-way ANOVA, *P < 0.05, **P < 0.01 vs. the NC inhibitor group or the MCS plus NC antagomir group; &&P < 0.01 vs. the NC inhibitor group or the ND plus NC antagomir group; #P < 0.05, ##P < 0.01 vs. the NC inhibitor plus MCD-sEVs group or the MCD plus NC antagomir group; $P < 0.05, $$P < 0.01 vs. the NC inhibitor plus HFD-sEVs group or the HFD plus NC antagomir group. **Figure S9.** Yield and size changes of hepatic sEVs between the two models. NAFLD or control hepatic sEVs were isolated by differential ultracentrifugation. (A) Protein concentration of hepatic sEVs was measured by BCA kit, n = 7–8 per group. (B) Representative images of sEV size distribution analysis by Flow Nanoanlyzer, n = 8 per group. (C) Concentration of hepatic sEVs was measured by Flow Nanoanlyzer, n = 8 per group. (D) Average size of hepatic sEVs was measured by Flow Nanoanlyzer, n = 8 per group. Data are expressed as the mean ±SEM. Statistics: One-way ANOVA, Ns: no significance, the MCD group were compared with the MCS group, and the HFD group were compared with the ND group. **Figure S10.** Genetic inhibition of NLRP3 suppresses NLRP3 inflammasome-associated microvascular hyperpermeability MVECs were transfected with 100 nmol/L NLRP3 siRNA (siNLRP3) or negative control siRNA (siNC) and incubated with or without 120 μg/mL NAFLD hepatic sEVs for 24 h. (A) Transfection efficiency of 5-FAM labeled siNLRP3. Scale bar, μm. (B) Representative western blot bands and the summarized data determined by densitometric analysis, n = 4 per group. (C-D) Representative flow cytometry images of caspase-1 FLICA staining and the summarized data of the positive cells, n = 4 per group. The fold changes were obtained by calculating the ratio of the positive cells of the treated groups to the siNC group. (E) Relative permeability of the endothelial monolayer to FITC-dextran, n = 4 per group. Data are expressed as the mean ± SEM. Statistics: One-way ANOVA, **P < 0.01 vs. siNC group; # P < 0.05, ## P < 0.01 vs. siNC plus MCD-sEVs group; &&P < 0.01 vs. siNC group; $$ P < 0.01 vs. siNC plus HFD-sEVs group. **Figure S11.** Effect of novel-miR-705 and novel-miR-1135 inhibitor on NLRP3 inflammasome activation. MVECs were transfected with 100nmol/L novel-miR-705 inhibitor or novel-miR-1135 inhibitor or negative control (NC) inhibitor and incubated with or without 120 μg/mL NAFLD hepatic sEVs for 24 h. (A-B) Representative flow cytometry images of caspase-1 FLICA staining and the summarized data of the positive cells, n = 4 per group. The fold changes were obtained by calculating the ratio of the positive cells of the treated groups to the NC inhibitor group. Data are expressed as the mean ± SEM. Statistics: One-way ANOVA, **P < 0.01 vs. NC inhibitor group; # P < 0.05 vs. NC inhibitor plus MCD-sEVs group; &&P < 0.01 vs. NC inhibitor group; Ns: no significance vs. NC inhibitor plus MCD-sEVs or HFD-sEVs groups. **Figure S12.** KEGG pathway enrichment analysis of the strong candidate genes. The common genes from cytokine activity and cytokine receptor binding signaling pathway with correlation scores ≥4 were considered as strong candidate genes of microvascular hyperpermeability. KEGG pathway enrichment analysis was performed to reveal the relevant signaling pathway of these strong candidate genes. **Figure S13.** Three-Dimensional cell model from the Z-stack obtained by stimulated emission depletion (STED) microscopy. MVECs were pretreated with or without 20 μmol/L dynasore for 40 min, followed by incubation of 120 μg/mL DiI-labeled hepatic sEVs for 8 h. Representative fluorescent confocal images of DiI-labeled hepatic sEVs (red), wheat germ agglutinin (WGA, green) with DAPI (blue). The pellet derived from the ultracentrifugation of DiI alone was used as a vehicle control. Scale bar, 10 μm.

**Additional file 2: Table S1.** Oligonucleotides. The synthesized oligonucleotides are listed in Table S1.

**Additional file 3: Table S2.** Raw reads of the top ten differentially expressed miRNAs from deep-sequencing. The raw data of the top ten differentially expressed miRNAs between MCS and MCD group from deep-sequencing are listed in Table S2. FDR: false discovery rate, FC: fold change.

## Data Availability

All data generated or analyzed during this study are included in this article and its Additional file.

## Abbreviations

AO: Acridine orange
AOI: Area of interest
CASP1: Caspase-1
CMD: Coronary microvascular dysfunction
FITC-dextran: Fluorescein isothiocyanate-conjugated dextran
GO: Gene Ontology
Hepatic sEVs: Small extracellular vesicles derived from the hepatic tissue
HFD: High-fat diet
HMEC-1: Human microvascular endothelial cell line-1
HMGB1: High mobility group box 1
HSCs: Hepatic stellate cells
HSP70: Heat shock protein 70
IL: Interleukin
LAMP1: Lysosomal associated membrane protein 1
LMP: Lysosomal membrane permeability
MCD: Methionine-choline-deficient
MCS: Methionine-choline-supplemented
MF: Molecular function
MiRNA: MicroRNA
MLVEC: Mouse lung microvascular endothelial cell
MR: Magic red
MVEC: Mouse vascular endothelial cell
NAFLD: Non-alcoholic fatty liver disease
NASH: Non-alcoholic steatohepatitis
NC: Negative control
ND: Normal diet
NLR: NOD-like receptor
NLRP3: NOD-like receptor family, pyrin domain containing 3
PA: Palmitic acid
SEVs: Small extracellular vesicles
SiNC: Negative control siRNA
SiNLRP3: NLRP3 siRNA
STED: Stimulated emission depletion
TLR: Toll-like receptor
TSG101: Tumor susceptibility hum 101
VWF: Von Willebrand factor
WGA: Wheat germ agglutinin
ZO: Zonula occludens
