# Supplementary material for: Hepatic small extracellular vesicles promote microvascular endothelial hyperpermeability during NAFLD via novel-miRNA-7
Source: J Nanobiotechnology. 2021 Nov 27;19:396. doi: 10.1186/s12951-021-01137-3 (PMC8626954; doi:10.1186/s12951-021-01137-3)

Supplementary Figure 1

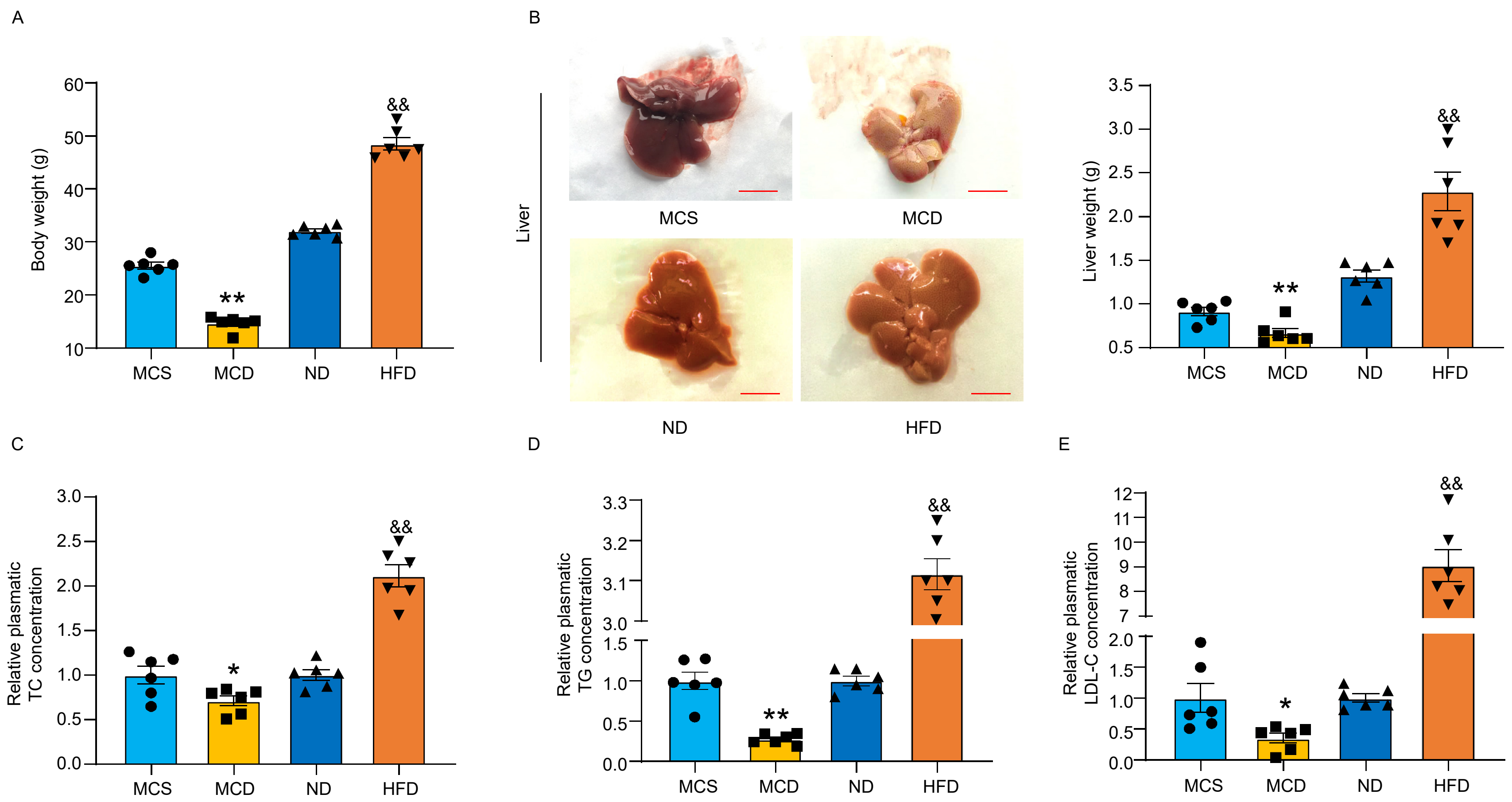

Supplementary Figure 2

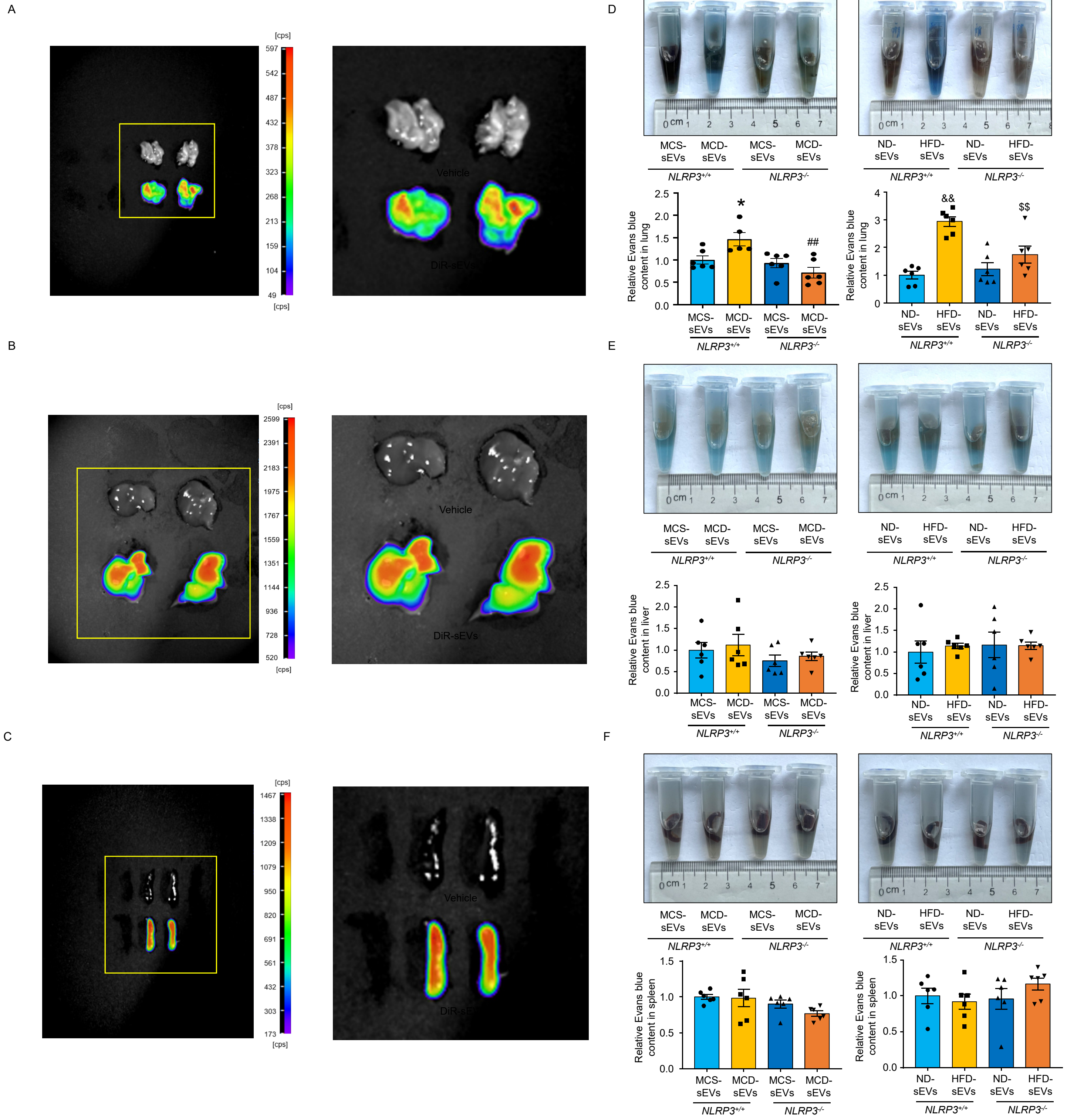

Supplementary Figure 3

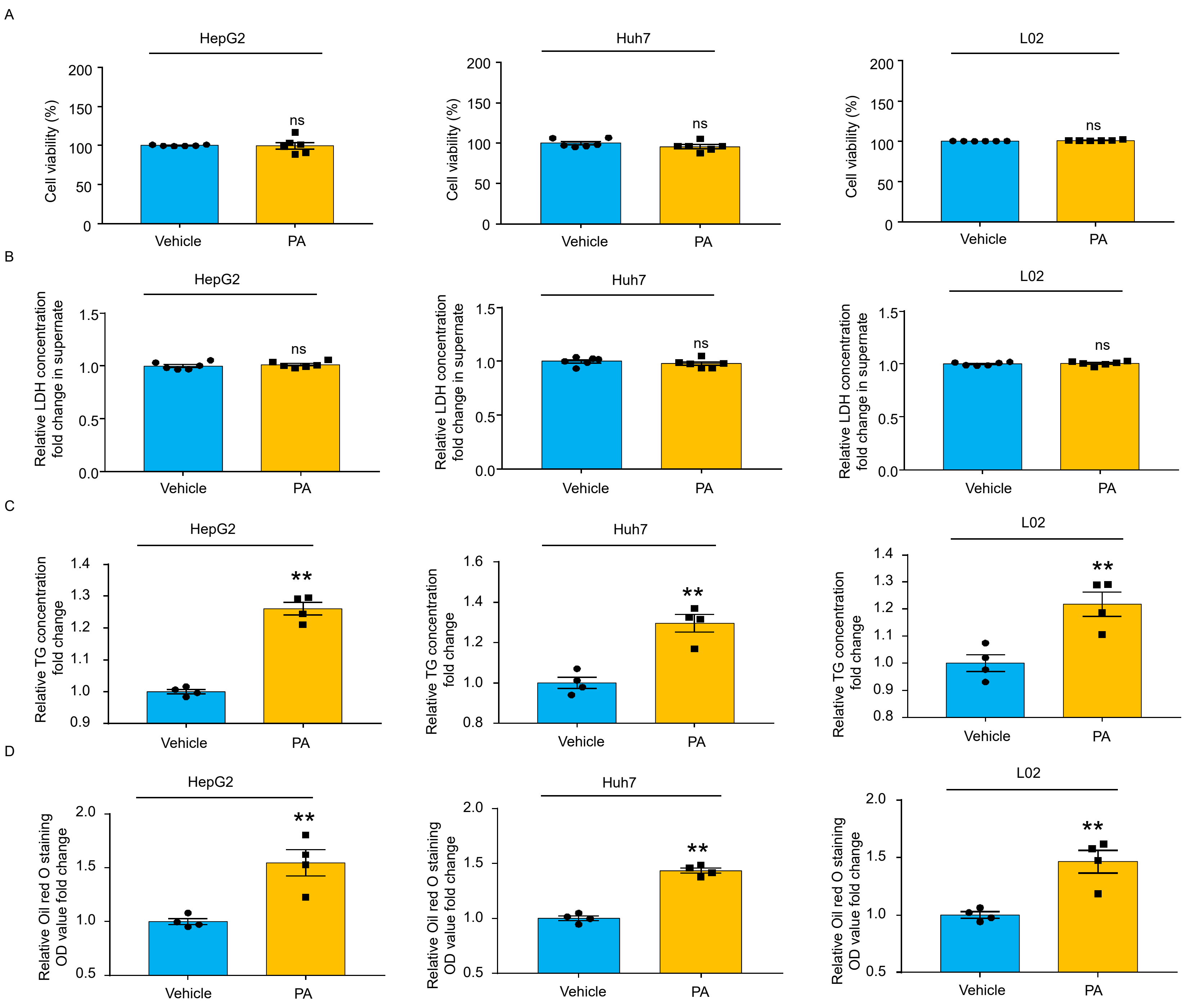

Supplementary Figure 4

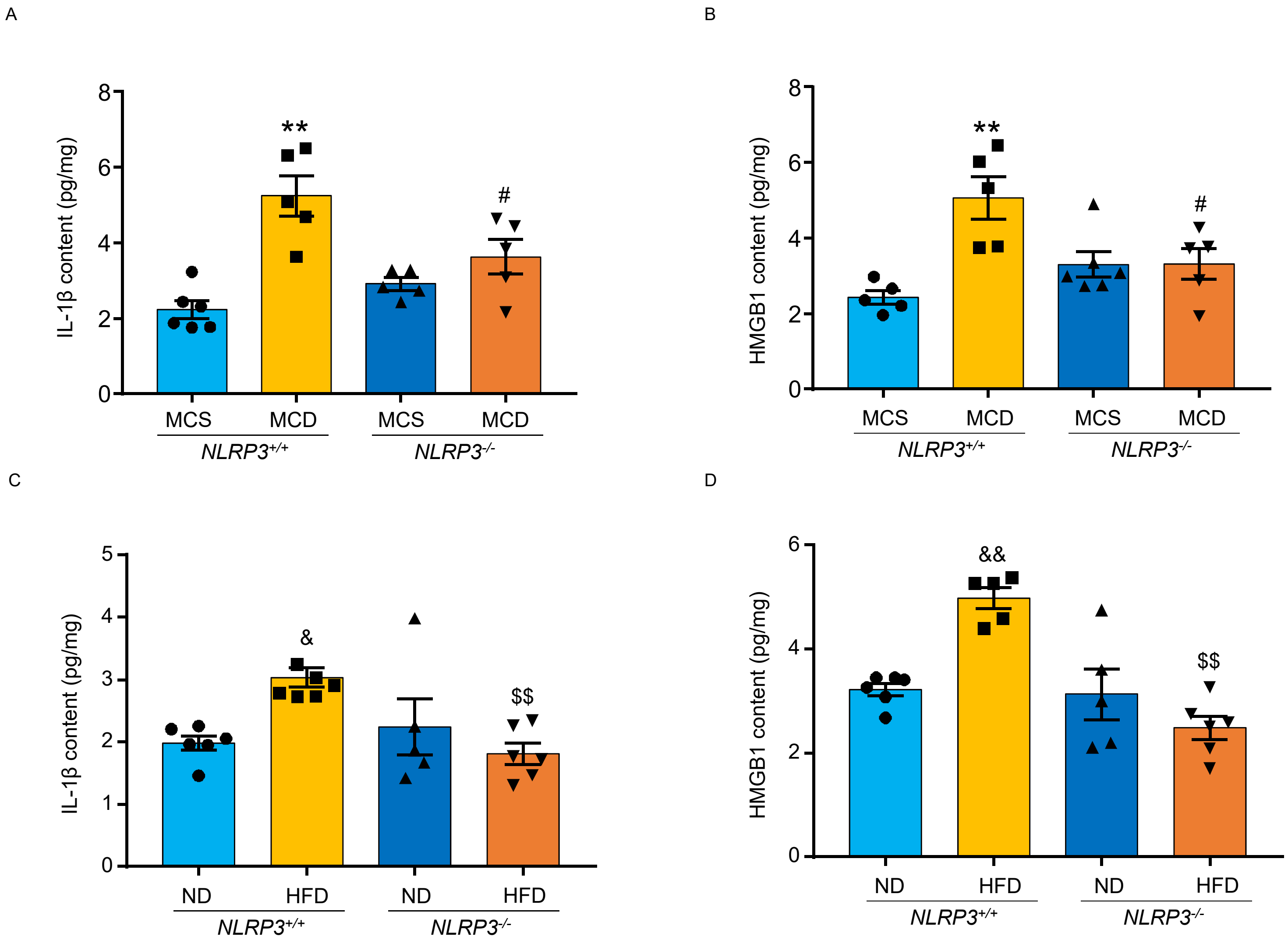

Supplementary Figure 5

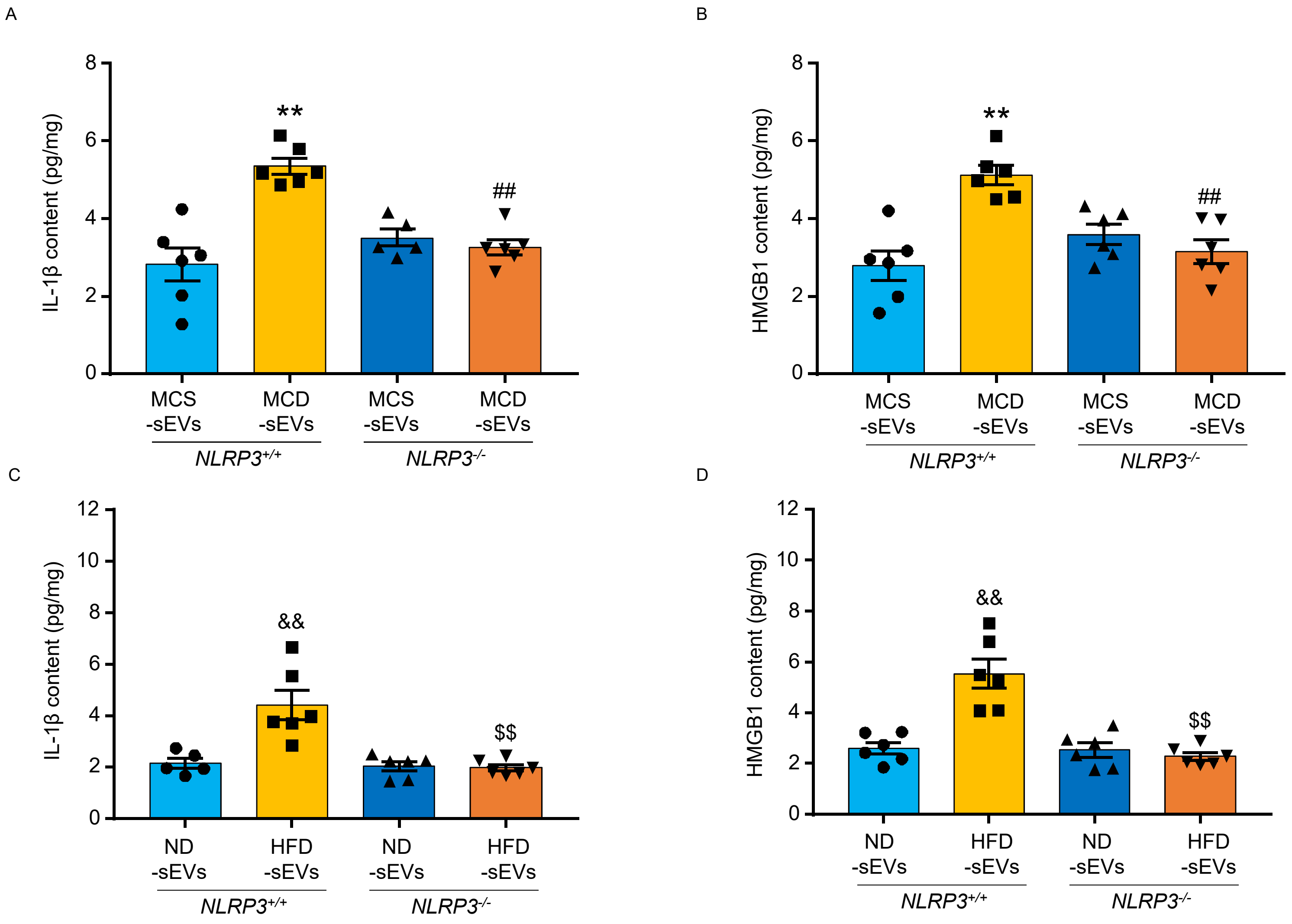

Supplementary Figure 6

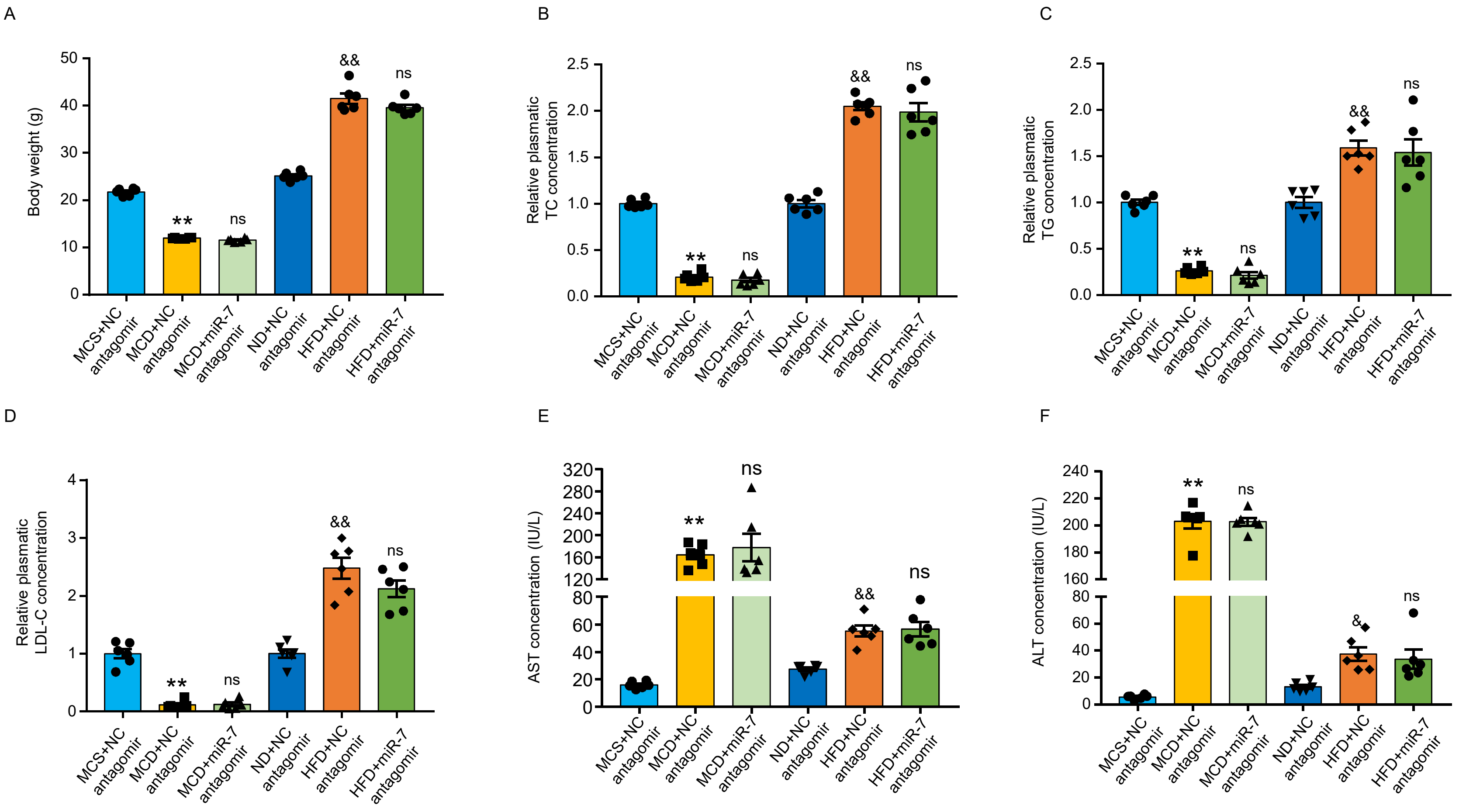

Supplementary Figure 7

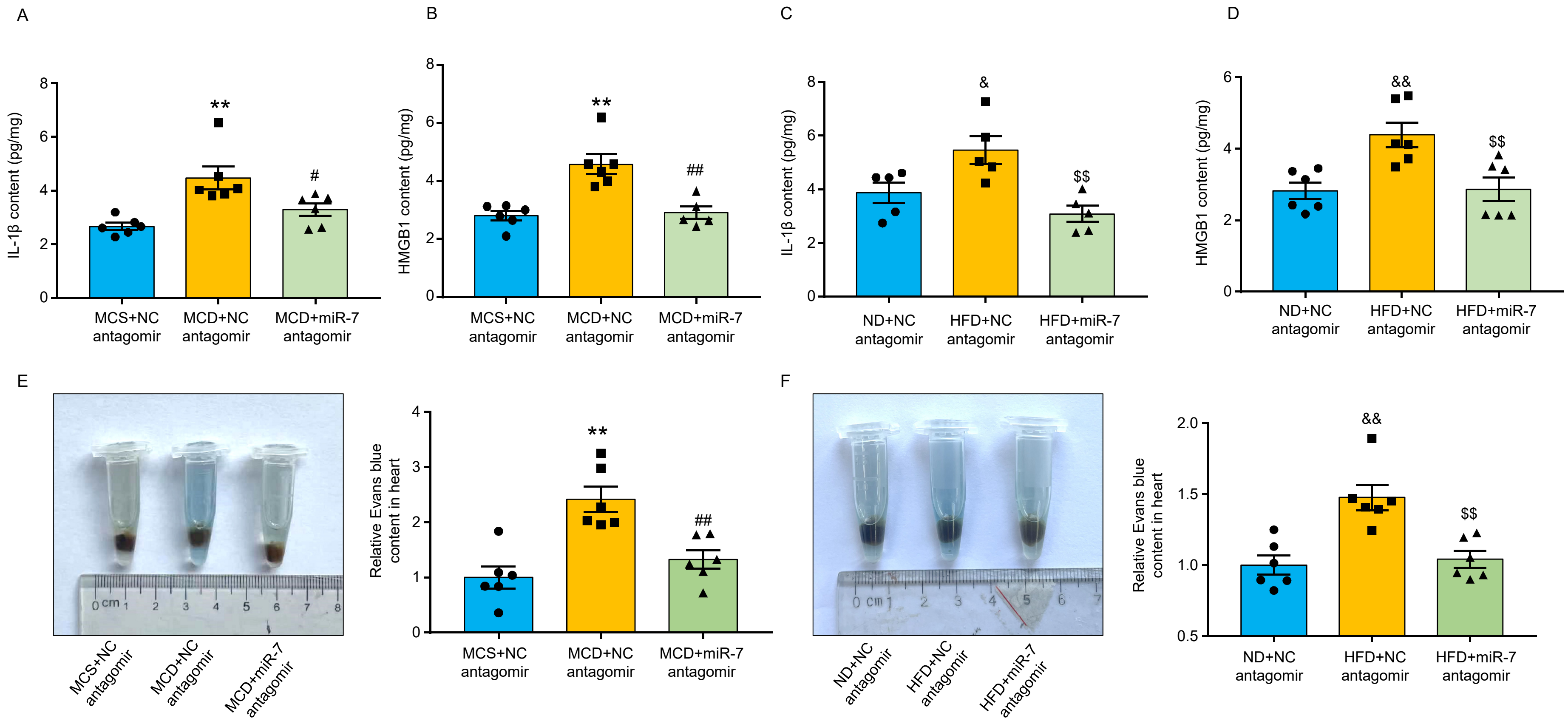

Supplementary Figure 8

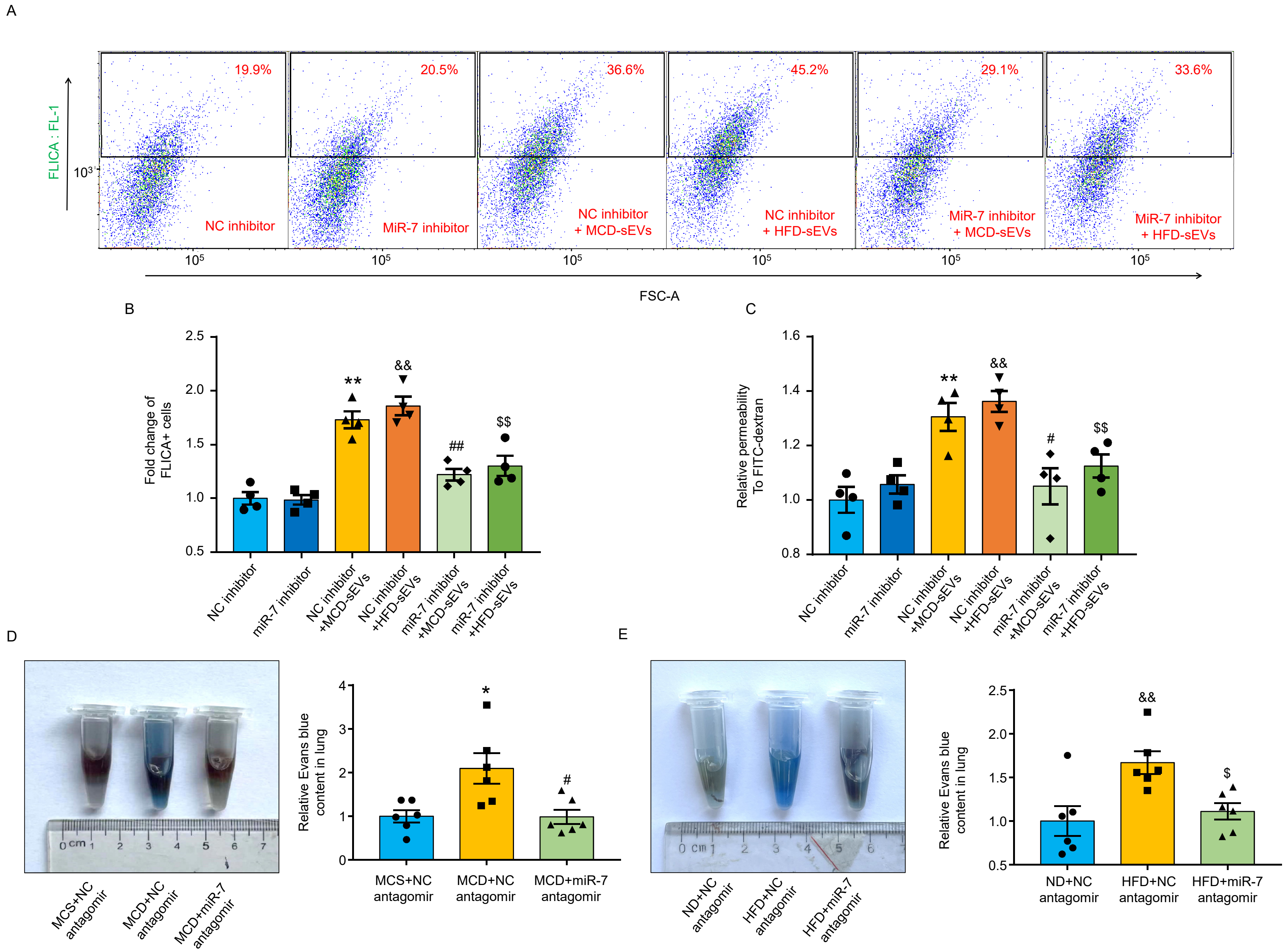

Supplementary Figure 9

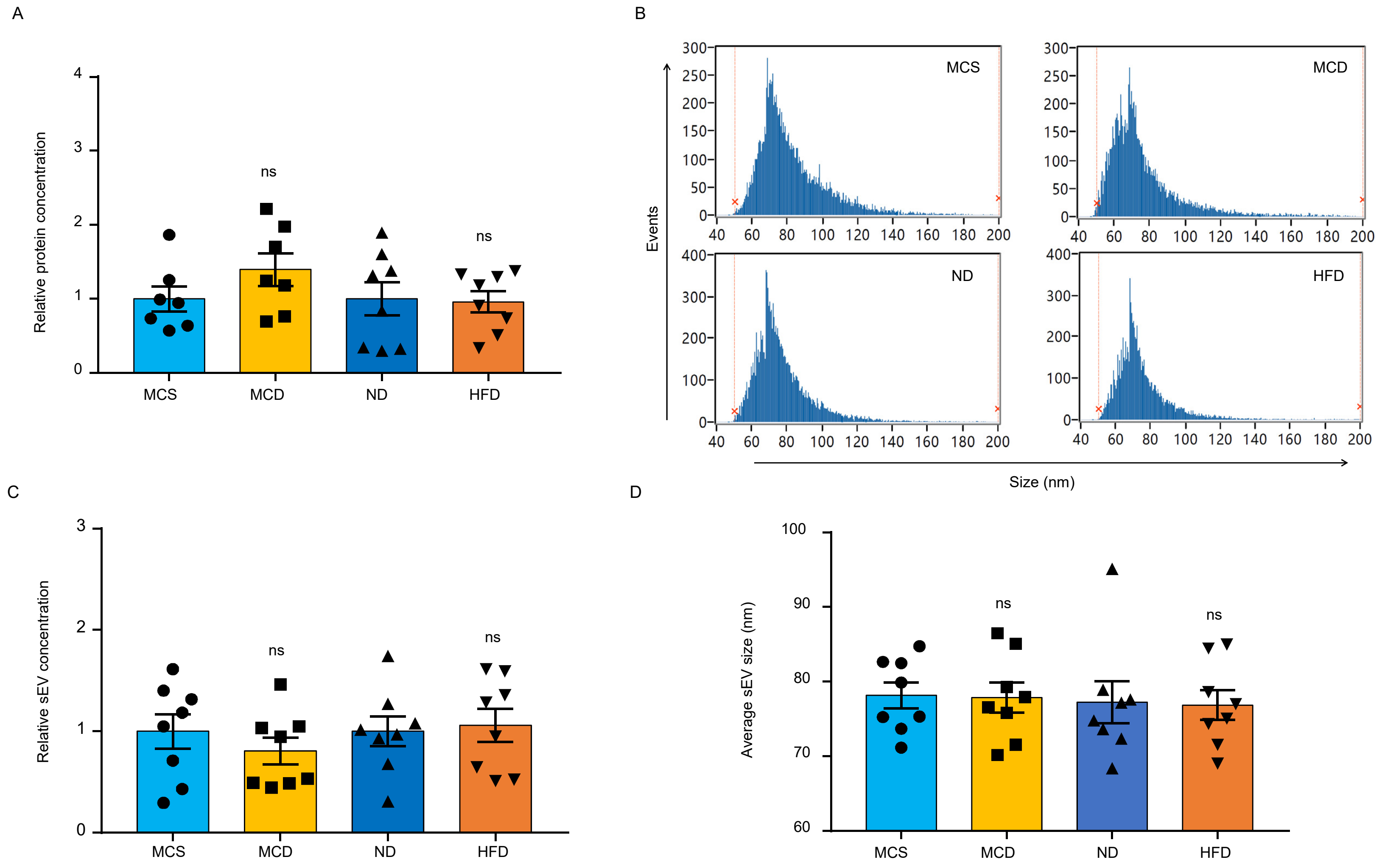

Supplementary Figure 10

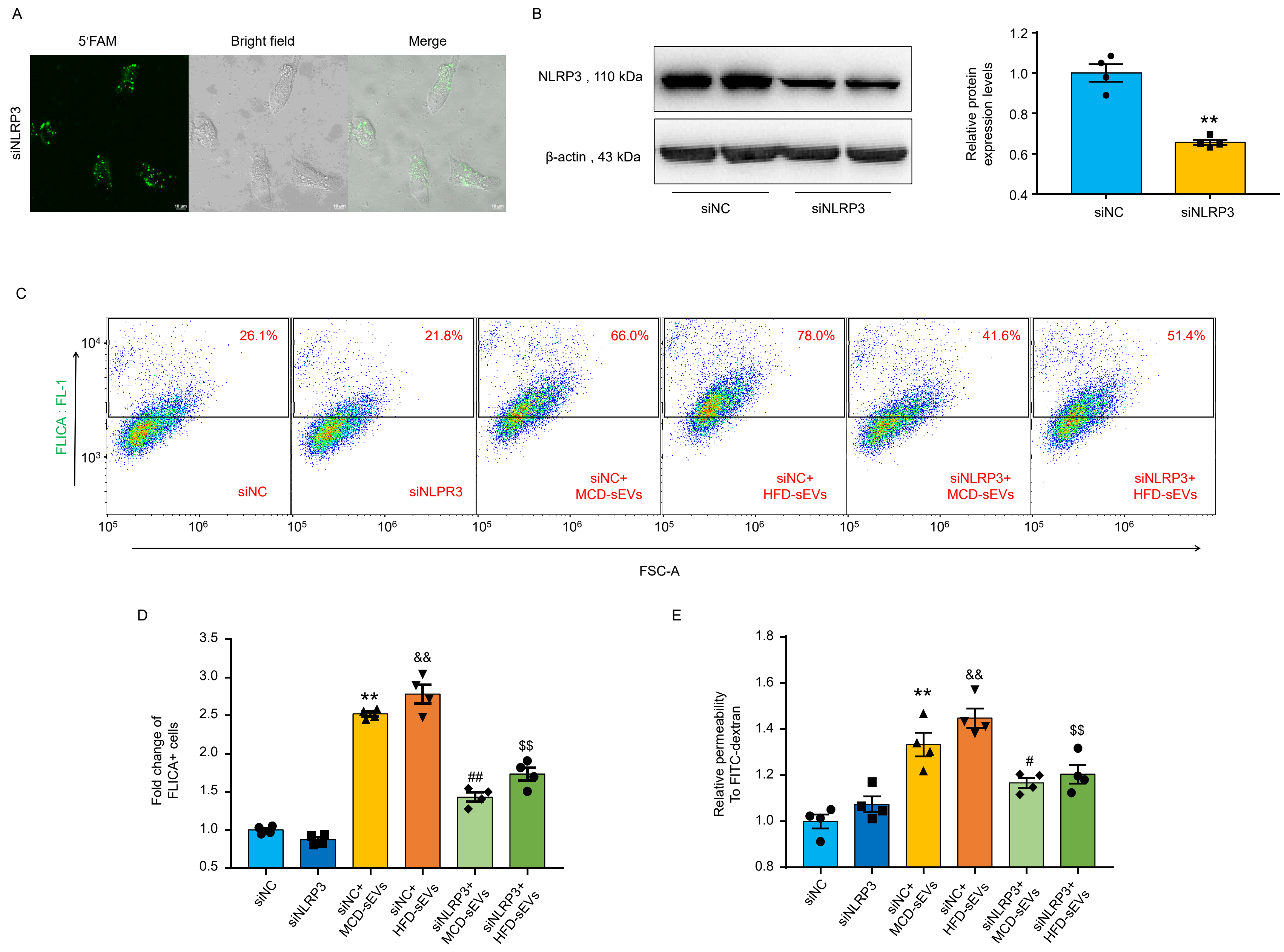

Supplementary Figure 11

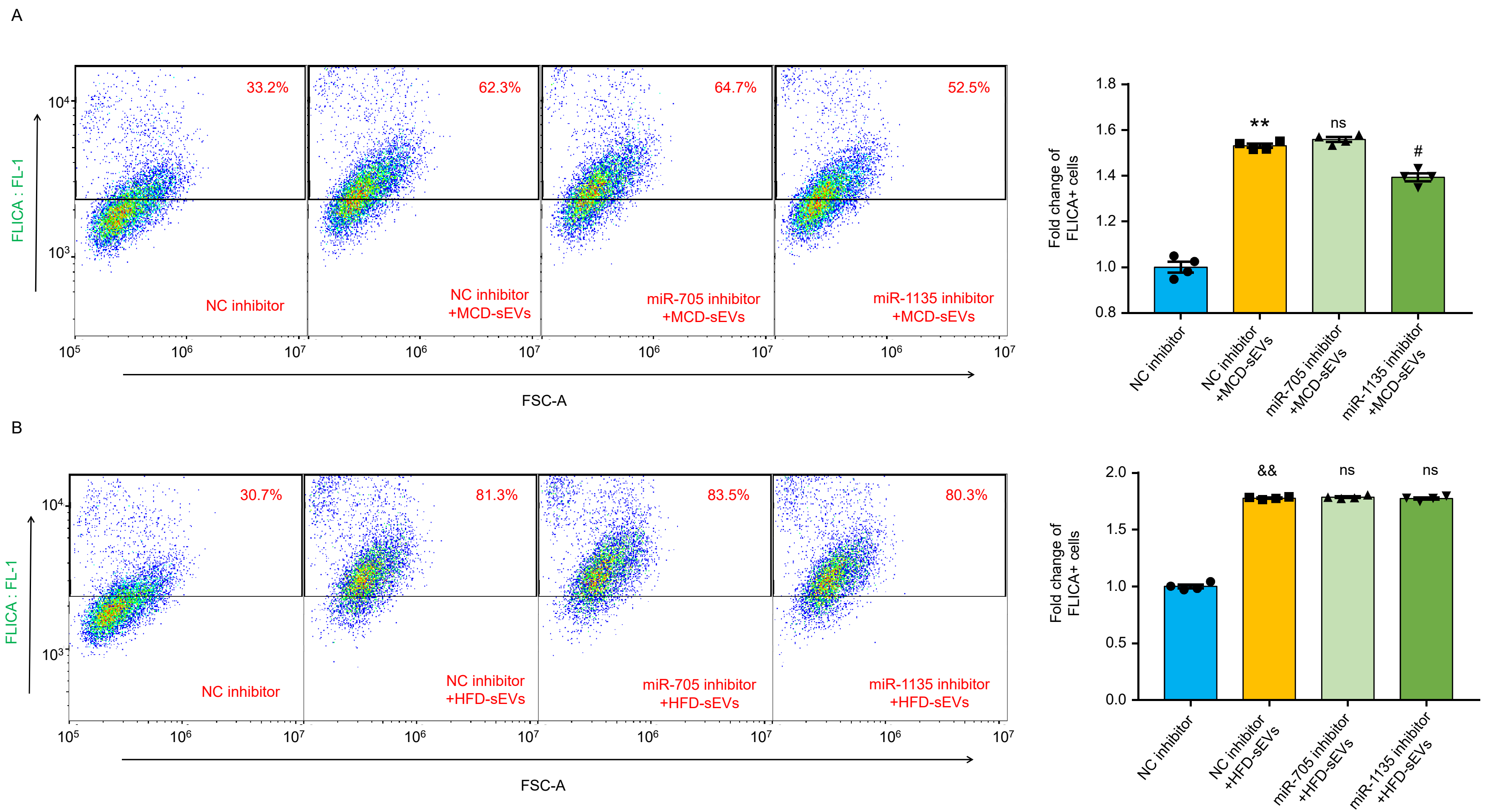

Supplementary Figure 12

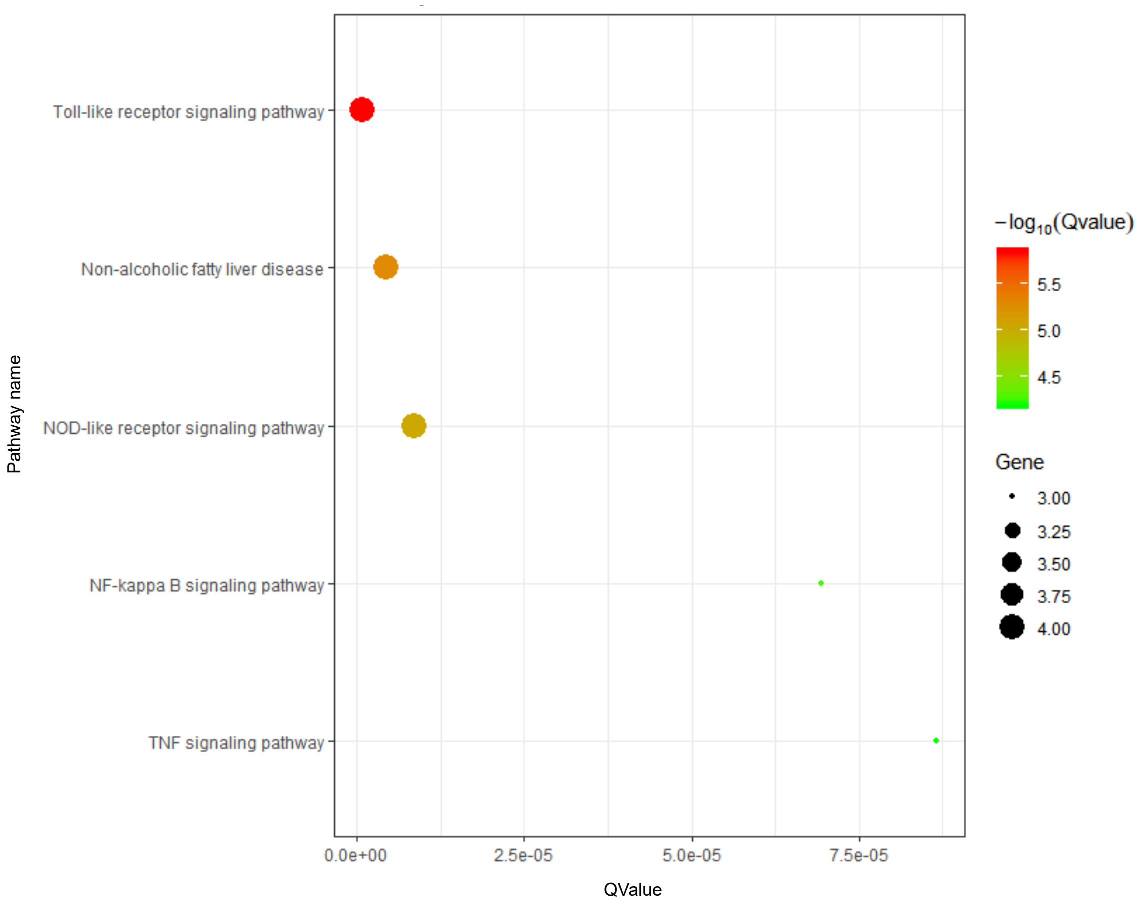

Supplementary Figure 13

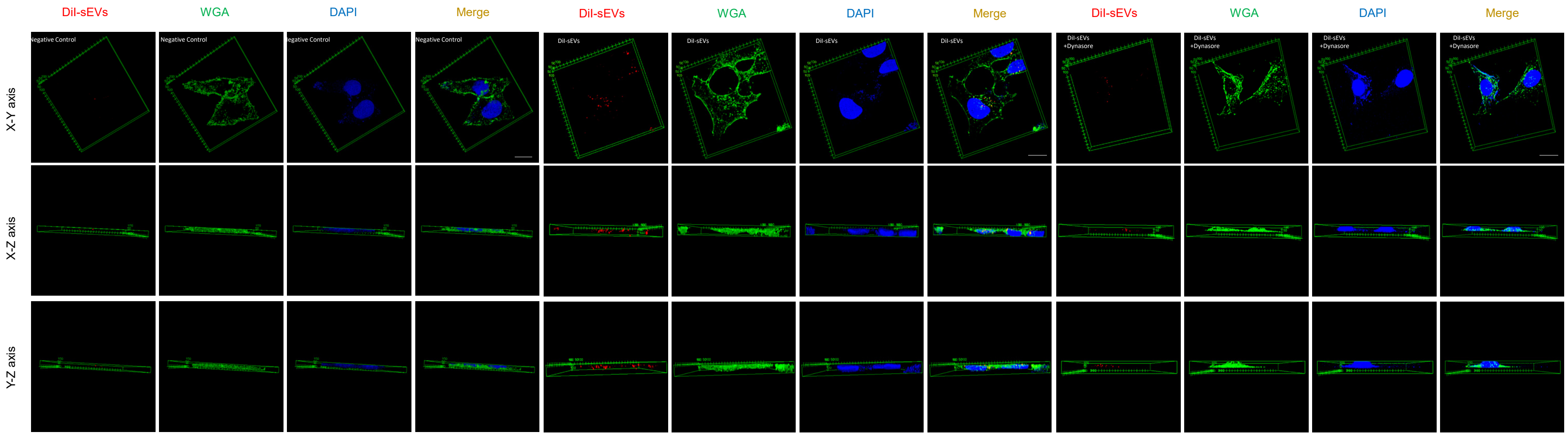

Supplement: Supplementary file 1 — Additional file 1: Figure S1. Body weight, liver weight and circulating lipid content in MCD and HFD-induced NAFLD mice. Mice were fed a MCD diet for 4 weeks or HFD for 16 weeks to induce NAFLD. (A) Body weight of NAFLD mice, n = 6 per group. (B) Representative images of liver and the liver weight of NAFLD mice, n = 6 per group. Scale bar:5 mm. (C-E) Relative plasma total cholesterol (TC), triglyceride (TG) and low-density lipoprotein cholesterol (LDL-C) content, n = 6 per group. Data are expressed as the mean ± SEM. Statistics: Student t-test, *P < 0.05, **P < 0.01 vs. the MCS group; &&P < 0.01 vs. ND group. Figure S2. Changes of microvascular endothelial permeability in lung, liver and spleen induced by hepatic sEVs. NAFLD or control hepatic sEVs were isolated by ultracentrifugation, identified, and administered to naive NLRP3+/+ and NLRP3-/- mice via caudal vein injection. (A-C) In vivo optical imaging system-obtained fluorescence imaging of lung, liver and spleen, n = 6 per group. The pellet derived from the ultracentrifugation of DiR alone was used as a vehicle control. (D-F) Representative images and the summarized data of Evans blue concentrations in lung, liver and spleen, n = 5-6 per group. Data are expressed as the mean ±SEM. Statistics: One-way ANOVA, *P < 0.05 vs. NLRP3+/+ mice injected with MCS hepatic sEVs; &&P < 0.01 vs. NLRP3+/+ mice injected with ND hepatic sEVs; ## P < 0.01 vs. NLRP3+/+ mice injected with MCD hepatic sEVs; $$ P < 0.01 vs. NLRP3+/+ mice injected with HFD hepatic sEVs. Figure S3. Cell viability, LDH leakage and lipid accumulation in steatotic hepatocytes. Human hepatocyte cell line HepG2, HuH7 and L02 were treated with 100 μmol/L palmitic acid (PA) or vehicle for 18 h. (A) Cell viability were measured using cell counting kit (CCK)-8, n = 6 per group. (B) Lactic dehydrogenase (LDH) leakage was estimated by measuring LDH activities in the cell culture supernatants, n = 6 per group. (C) Intracellular triglyceride (TG) contents were meas [file 12951_2021_1137_MOESM1_ESM.pdf]
