# Supplementary material for: Hepatic small extracellular vesicles promote microvascular endothelial hyperpermeability during NAFLD via novel-miRNA-7
Source: J Nanobiotechnology. 2021 Nov 27;19:396. doi: 10.1186/s12951-021-01137-3 (PMC8626954; doi:10.1186/s12951-021-01137-3)
Supplement: Supplementary file 2 — Additional file 2: Table S1. Oligonucleotides. The synthesized oligonucleotides are listed in Table S1. [file 12951_2021_1137_MOESM2_ESM.pdf]

Supplementary Table 1. Oligonucleotides

novel-miR-7 stem-loop primer	5′-GTCGTATCCAGTGCAGGGTCCGAGG TATTCGCACTGGATACGACCACTGA-3′
novel-miR-7 primer	F: 5′- CGAGGGGCTGGAGAGATAGC-3′<br>R: 5′- AGTGCAGGGTCCGAGGTATT-3′
U6 stem-loop primer	5′-GTCGTATCCAGTGCAGGGTCCGAGG TATTCGCACTGGATACGACAAAATA-3′
U6 primer	F: 5′- AGAGAAGATTAGCATGGCCCCTG -3′<br>R: 5′- ATCCAGTGCAGGGTCCGAGG -3′
novel-miR-7 mimic	F: 5′-AGGGGCUGGAGAGAUAGCUCAGUG-3′<br>R: 5′-CUGAGCUAUCUCUCCAGCCCCUUU-3′
negative control mimic	F: 5′-UUCUCCGAACGUGUCACGUTT-3′<br>R: 5′-ACGUGACACGUUCGGAGAATT-3′
novel-miR-7 inhibitor/<br>novel-miR-7 antagomir	5′-CACUGAGCUAUCUCUCCAGCCCCU-3′
negative control inhibitor/<br>negative control antagomir	5′-CAGUACUUUUGUGUAGUACAA-3′
pGL3-LAMP1-3’UTR plasmid	F: 5′-GCTCTAGAAGCCTGGTGGGCAGG-3′<br>R: 5′-GCTCTAGAGACAGACAAAGAAGGAGACTT-3′
NLRP3 siRNA	F: 5′-GGUUCUGAGCUCCAACCAUTT-3′<br>R: 5′-AUGGUUGGAGCUCAGAACCTT-3′
negative control siRNA	F: 5′-UUCUCCGAACGUGUCACGUTT-3′<br>R: 5′-ACGUGACACGUUCGGAGAATT-3′
novel-miR-705 inhibitor	5′-AGCUCCACCACUGAGCUACAUCCCC-3′
novel-miR-1135 inhibitor	5′-GCCAACAUCUUCCAAGAGCAUCUCU-3′
