# Supplementary material for: Hepatic small extracellular vesicles promote microvascular endothelial hyperpermeability during NAFLD via novel-miRNA-7
Source: J Nanobiotechnology. 2021 Nov 27;19:396. doi: 10.1186/s12951-021-01137-3 (PMC8626954; doi:10.1186/s12951-021-01137-3)
Supplement: Supplementary file 3 — Additional file 3: Table S2. Raw reads of the top ten differentially expressed miRNAs from deep-sequencing. The raw data of the top ten differentially expressed miRNAs between MCS and MCD group from deep-sequencing are listed in Table S2. FDR: false discovery rate, FC: fold change. [file 12951_2021_1137_MOESM3_ESM.pdf]

Supplementary Table 2. Raw reads of the top ten differentially expressed miRNAs from deep-sequencing

MicroRNA	MCS_01	MCS_02	MCS_03	MCD_01	MCD_02	MCD_03	FDR*	log2FC#
novel_miR_7	0	7	1	137	123	168	4.30E-26	5.952173
novel_miR_2084	0	0	1	5	12	12	0.004957	5.047025
novel_miR_1135	2	1	1	33	29	39	1.42E-11	4.847630
mmu-miR-27a-5p	0	1	0	6	6	13	0.000228	4.820164
novel_miR_830	1	0	0	8	8	7	0.000485	4.777667
novel_miR_1069	0	1	0	10	4	9	0.000494	4.680046
novel_miR_705	3	1	0	25	16	50	1.69E-06	4.626626
novel_miR_1383	0	0	1	4	7	7	0.003439	4.348673
novel_miR_2352	0	0	1	3	4	12	0.018606	4.284491
novel_miR_292	0	1	0	7	2	8	0.005338	4.196108

*FDR: false discovery rate, #FC: fold change
